# Assessing Cobalt(II/III) Complex Purity Using XRD and Its Impact on Effectiveness of Catalytic Chain Transfer Polymerization

**DOI:** 10.1021/acs.macromol.5c00049

**Published:** 2025-03-06

**Authors:** Xiaofan Yang, Jie Liu, Nicholas R. Bagnall, Johan P. A. Heuts, Brady Worrell, Christopher Waldron, David M. Haddleton

**Affiliations:** † Department of Chemistry, University of Warwick, Coventry CV4 7AL, U.K.; ‡ Department of Physics, University of Warwick, Coventry CV4 7AL, U.K.; § Department of Chemical Engineering & Chemistry and Institute for Complex Molecular Systems, Eindhoven University of Technology, P.O. Box 513, MB Eindhoven 5600, The Netherlands; ∥ Department of Chemistry & Biochemistry, University of Denver, Denver, Colorado 80210, United States; ⊥ Research and Technology Platforms, University of Warwick, Coventry CV4 7AL, U.K.

## Abstract

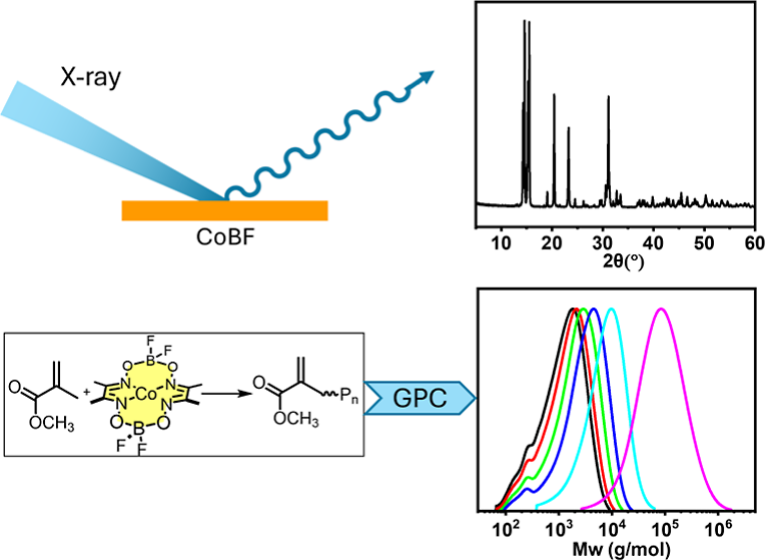

Catalytic chain transfer polymerization (CCTP) of methacrylates using Co(II)/Co(III) catalysts has been established for over 30 years. These catalysts have been used in commercial products in diverse markets including automotive coatings, printing and dental composites. However, it has proved difficult routine and reliable tests of catalyst purity that indicate the activity and hence the amount of catalyst required to achieve a desired product. The catalysts are difficult to characterize due to being paramagnetic, sparingly soluble and highly symmetrical. This work set out to first determine the structure of “impurities” that might be present after synthesis or might form over time. Using this knowledge the work then used powder X-ray spectra and the deconvolution of obtained spectra to the known structures to obtain a measure of catalyst purity and activity. This was then correlated with the chain transfer constants from Mayo plots as a measure of activity. We discovered some new hitherto unknown cobalt structures and obtained structural data and developed a deconvolution algorithm that extracts the composition of the catalyst mixtures to give compositional data for 6 cobalt-containing compounds as well as starting materials and inorganic salts. Three of the cobalt species were shown to be active and thus the composition data was shown to be a measure of catalyst activity as tested against polymerization data. Catalyst samples from two laboratories were obtained which had been prepared at different times as well as a commercial sample. The powder X-ray method developed was subsequently used to validate the catalyst activity toward CCTP of methyl methacrylate. Thus, a relatively simple powder X-ray measurement on a catalyst sample can be used as a direct measure of activity and purity.

## Introduction

**Figure 1:**
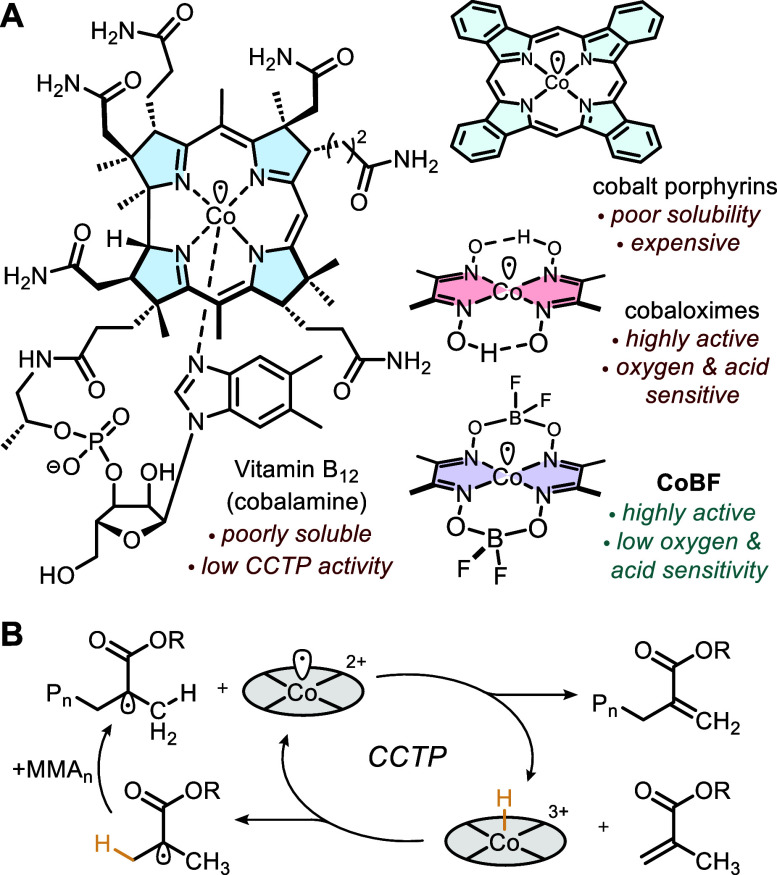
(A) Cobalt complexes as CCTP catalysts. The chemical structure of CoBF is the BF_2_ bridged cobaloxime. (B) Schematic of the CCTP reaction cycle.

Catalytic chain transfer polymerization (CCTP) is an efficient and well-established method to produce low molecular weight vinyl-terminated polymethacrylates. This has been the focus of some excellent reviews from both historical and technical perspectives.^1−7^ CCTP (Figure 1b) utilizes certain cobalt(II) complexes as chain transfer agents (CCTAs) that act by abstraction of a hydrogen from a methacrylic propagating radical chain to give a vinyl terminated oligomeric/polymer product and an intermediate cobalt(III) hydride. The intermediate Co[III]–H presumably reinitiates polymerization via the transfer of a hydrogen atom to monomer (chain transfer) regenerating the active Co(II) species resulting. Therefore, only small quantities of the CTA, relative to the monomer, are required to significantly reduce the molecular weight of the product. Indeed, the observed chain transfer constants (*C*_s_) in this reaction are usually over 4 orders of magnitude higher than thiols which are often used in commercial chain transfer, which in turn have chain transfer constants up to 6 orders of magnitude higher than common hydrocarbons, for example.

Smirnov and co-workers first noticed the effect in the mid-1970s when they added a range of different metal porphyrin complexes to the free radical polymerization of methyl methacrylate and other vinyl monomers. They found that even small amounts of porphyrin complexes acted as very efficient chain transfer agents, especially in the case of methacrylates resulting in significant lowering of the resultant molecular weights.^8−11^ Wayland used organocobalt(III) porphyrin complexes for the living radical polymerization of acrylates. The addition of a small amount of these complexes resulted in a linear increase in the monomer conversion, the number-average molecular weight being directly proportional to the [Co]/[monomer] together with a relatively narrow molecular weight dispersity indicating an efficient living radical polymerization process.^12,13^ CCTP was subsequently introduced into the Western world by the Glidden company, which reported the use of cobalt(II) dimethyldioxime to lower the molecular weight of poly(methyl methacrylate) (PMMA) and PMMA copolymers.^14^ It is noted that these catalysts have a similar structure and reaction profiles to Vitamin B_12_ and many catalysts effective for CCTP have also been studied as Vitamin B_12_ model compounds. In 1984, cobalt(II) dimethylglyoxime (cobaloxime) was employed by O’Driscoll being used in a continuous process for the production of kilogram quantities of low molecular weight poly(methyl methacrylate) (PMMA).^15^ These vinyl functional low molecular weight oligomers were synthesized in a tubular reactor and isolated from unreacted monomers using devolatilization in an extruder.^15^ However, despite the scalability of CCTP certain cobaloxime(s) are sensitive to oxygen and prone to acidic hydrolysis leading to the formation of inactive cobalt complexes and loss of activity. In order to circumvent this issue, Janowicz of DuPont, reported the use of a BF_2_ bridged complex which stabilizes the complex against acidic hydrolysis with the catalyst remaining active for many hours at temperatures of up to 80 °C, even at pH < 2.^16^ It is noted that this BF_2_ bridged complex was first reported by Bakac and Espenson^17^ as a stable inorganic complex as the unbridged complex decomposes almost instantaneously to Co(II) at low pH. This and related complexes were studied in attempts to further understand the mechanism of vitamin B_12_.^18^ The methyl substituent groups on the ring of the BF_2_ bridged cobaloxime (CoBF) can be changed to other alkyl, phenyl and substituted phenyl groups which result in a wide range of different solubilities in organic and aqueous media with little effect on the activity of the [Co(II)]/[Co(III)]-R in low viscosity reactions, Figure 1. Thus, this has allowed for this chemistry to be developed and exploited commercially in suspension, emulsion, solution and bulk polymerization processes.^19−27^ CCTP is effective in all evaluated free radical polymerization processes including mini-emulsion, emulsion, in supercritical carbon dioxide, solution and bulk.^23,26−30^ CCTP can be applied to produce low molecular weight vinyl terminated polymers such as in latex particles for dyes, pigments or colorants; haircare products to protect hair from environmental damage; synthesis of branched or graft polymers or macromonomers for hydrogels.^31^

The high efficiency of CCTP even allows for multifunctional methacrylates to be polymerized without observing cross-linking to vinyl functional hyperbranched polymers^32−34^ in bioconjugation chemistry,^35,36^ and to make block copolymers^37^ by sulfur-free reversible addition–fragmentation transfer (RAFT) polymerization.^36,38,39^ More recently, CCTP has been used by the 3 M Company to produce difunctional addition–fragmentation monomers (AFMs) which have been exploited in commercial dental resins to relieve stress during photopolymerization finding widespread and global use.^40−43^ These difunctional cross-linking agents containing an addition–fragmentation site have been exploited for similar effects in vat photolithographically 3D printing.^44,45^

CoBF is the most commonly used catalyst, especially in academic laboratories. Even though CoBF has been used in a range of commercial products for more than 30 years, catalyst purity and activity over time have been proven difficult to assess. As CoBF is only sparingly soluble in organic solvents and aqueous media, purification by chromatography is difficult and not scalable. Furthermore, CoBF is paramagnetic which precludes the use of NMR or other related techniques to aid in determining purity. Indeed, the most common technique used to determine the activity of a given batch of CoBF is to calculate the chain transfer constant (*C*_s_). This laborious process is accomplished by adding different concentrations of CoBF to MMA and free-radically polymerizing them to a low conversion (<10%). If the *C*_s_ value obtained by this method is above a certain threshold (*C*_s_ > 10^3^), then the catalyst in question is considered “*active*”.^46,47^

In 2023, the Worrell lab reported the use of CCTP in bulk photopolymerizations to alter the mechanical properties of the resultant cross-linked materials.^46^ Here, CoBF was found to be the optimal CCTP catalyst; it could significantly reduce the glass transition temperature (*T*_g_) and storage modulus at ppm quantities. CCTP is increasingly being used in a range of photopolymerization reactions and Worrell discussed the problems in detail faced with determining catalyst purity and thus calculating the amounts required to reach the desired product. They discussed in detail the issues related to determining catalyst purity and the requirement to have a highly active catalyst to observe changes in the mechanical performance of the photopolymers. To quantify the purity of CoBF, a UV/vis method was developed in tandem with (spectroelectrochemistry) SEC to correlate absorbance spectra and *C*_s_ values, respectively. The described method uses the ratio of absorbance peaks at 425 and 322 nm (Abs_425_/Abs_322_) where they empirically found that the higher the ratio, the more active the CoBF. Despite this, determining the absolute purity and activity of a given batch of CoBF remains an unmet challenge. Furthermore, as CoBF and related complexes find applications outside of CCTP in energy^48^ and complex small molecule synthesis,^49^ developing reliable synthetic procedures and understanding the identity and role of inorganic byproducts has become a critical issue.^46,47^

**Figure 2:**
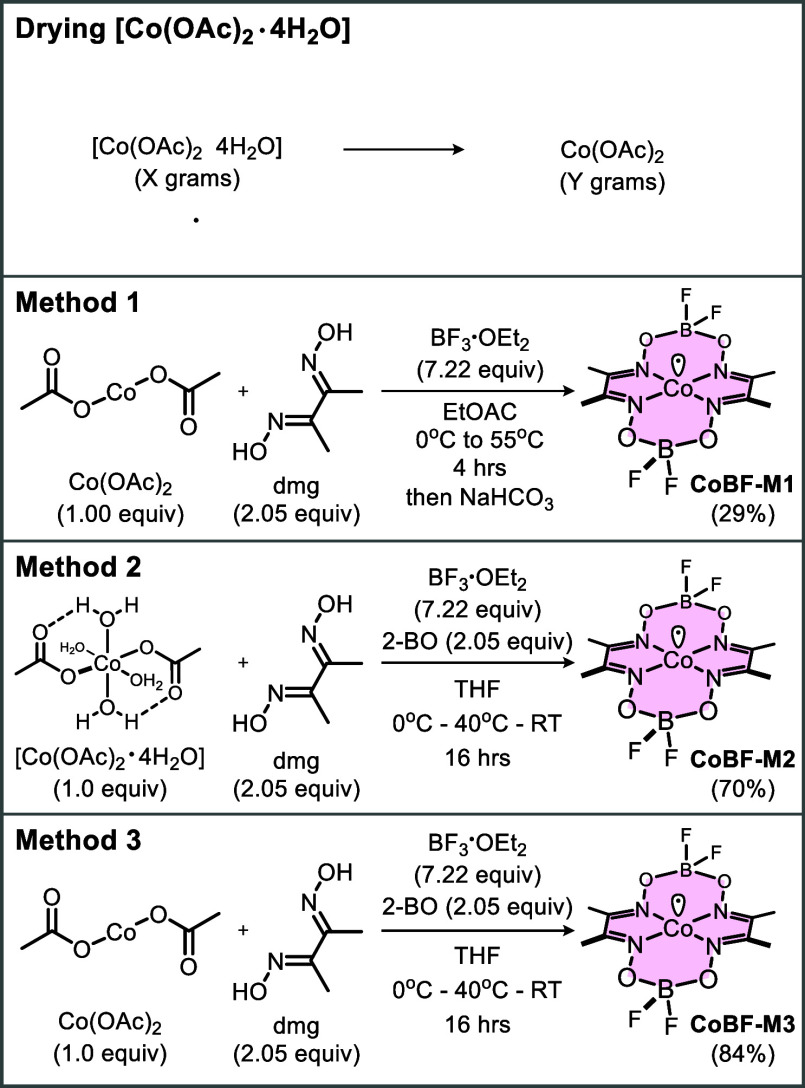
Three different methods of CoBF synthesis.

Herein, we obtained the single crystal structures of some compounds produced during the synthesis of CoBF.^50^ The powder X-ray diffraction data were recorded for these samples from synthesized CoBF (by methods 1, 2 and 3, Figure 2), samples that had been stored over several years in different laboratories and conditions as well as a commercial sample from a fine chemical supplier. Subsequently, the phase identification and quantitative analysis of the XRD patterns were performed to give a measure of the purity (active wt %) of each sample. The results demonstrated that composition varied among these catalyst samples synthesized by different methods and stored at under different conditions. The chain transfer constant (*C*_s_) of CoBF was determined from a conventional Mayo plot from GPC data.^51^ The correlation between the weight/mol amount of the effective catalyst components in the catalyst samples and the *C*_s_ values was evaluated. Finally, we obtained samples from Worrell’s laboratory in the US, where CoBF is currently being actively used, and legacy samples from Heuts’ laboratory in The Netherlands together with the CoBF synthesized at our lab (UK) over many years to investigate any correlation between the purity of catalyst obtained through the refinement of XRD patterns and the measured polymerization chain transfer activity.

## Materials and Methods

Methyl methacrylate (MMA, 99% (GC), ≤30 ppm MEHQ as inhibitor) and 2,2′-azobis(2-methylpropionitrile) [AIBN, ≥98% (GC)] were purchased from Sigma-Aldrich (UK). Cobalt(II) acetate tetrahydrate (98%) was purchased from ABCR; dimethylglyoxime (DMG) (ACS reagent, ≥99%) and 2-butanone oxime (99% (GC)) were purchased from Sigma-Aldrich (UK). Boron trifluoride diethyl etherate (BF_3_–OEt_2_) was purchased from Fluka Analytical. All bulk solvents: THF, toluene, water, methanol, and diethyl ether were purchased from Sigma-Aldrich and used as received.

Samples CoBF 1998, CoBF 1999 and CoBF Ark were previously prepared at the University of Warwick between 1998 and 2018. Samples of CoBF (using methods 1–3 illustrated below) were synthesized in 2024 using these three different methods. Samples CoBF 09/03, CoBF 09/06 and CoBF10/01 were synthesized by Hans Heuts’ group (Eindhoven University of Technology, Netherlands) more than 10 years earlier made by the method reported by Suddaby^19^ and stored in a vial on a bench. Samples BW CoBF1 and BW CoBF2 were recently made by Brady Worrell’s group (University of Delaware) who also supplied the commercial Strem sample (Strem CoBF) purchased in 2021.

### Preparation of CoBF

#### Method 1

Water of hydration of cobalt(II) acetate tetrahydrate was removed in a vacuum oven at 110 °C overnight, with the solid changing from pale pink crystallites to a purple powder, indicating the formation of anhydrous cobalt(II) acetate.

Anhydrous cobalt(II) acetate (3.5 g, 19.7 mmol, 1.00 mol equiv) and dimethylglyoxime (4.69 g, 40.2 mmol) were added to a 250 mL three-neck round-bottom flask. The solid mixture was then deoxygenated for 1 h by flushing the flask with nitrogen. Following this, deoxygenated ethyl acetate (100 mL) was added into the flask to form a suspension which was allowed to stir in an ice bath for 30 min. Subsequently, boron trifluoride diethyl etherate (17.5 mL, 142.2 mmol, 7.22 equiv) was added dropwise via a deoxygenated syringe over 30 min. The flask was then removed from the ice bath and heated at 55 °C for 1 h. A brown precipitate formed suspended in a dark brown solution during this period, the flask was then cooled to ambient temperature. Sodium bicarbonate (5.1 g, 60.7 mmol, 3.1 equiv) was added in portions to the solution for neutralization. The solution was cooled in an ice bath for 1 h and then filtered by suction. The filtered solid was washed with water (70 mL × 2) and then methanol (50 mL × 2).

The purification of the crude product was carried out by adding the product to methanol with 6 times equivalent mass, the mixture was sonicated for 5 min and then stirred for 30 min followed by filtration by suction. The purification was repeated twice to obtain higher product quality at the expense of the yield (2.23 g, 29.4%).

#### Method 2

Cobalt(II) acetate tetrahydrate (4.9 g, 19.7 mmol) and dimethylglyoxime (4.69 g, 40.2 mmol) were placed in a 250 mL three-neck round-bottom flask with a magnetic stirring bar and purged with nitrogen for 1 h. Deoxygenated THF (90 mL) was then added to the three-neck round-bottom flask. The mixture was vigorously stirred. Deoxygenated 2-butanone oxime (3.5 g, 40.2 mmol) was then added to the flask. The mixture was cooled to 0 °C in an ice bath. Boron trifluoride diethyl etherate (17.5 mL, 142.2 mmol) was added dropwise with a deoxygenated syringe over 30 min. The solution was stirred and heated to 40 °C for 2 h. The resulting mixture was cooled to ambient temperature and left overnight. It was subsequently filtered and washed with water (50 mL × 2), diethyl ether (30 mL × 2) and finally methanol (30 mL × 2) to collect the crude product. The same purification steps as mentioned in method 1 were carried out after the crude product was collected (5.31 g, 69.8%).

#### Method 3

Anhydrous cobalt(II) acetate (3.5 g, 19.7 mmol) was used in place of cobalt(II) acetate tetrahydrate, the other steps were the same as for method 2. Purification processes were carried out twice after filtration (6.38 g, 83.9%).

### Crystallization of Obtained Products

**Figure 3:**
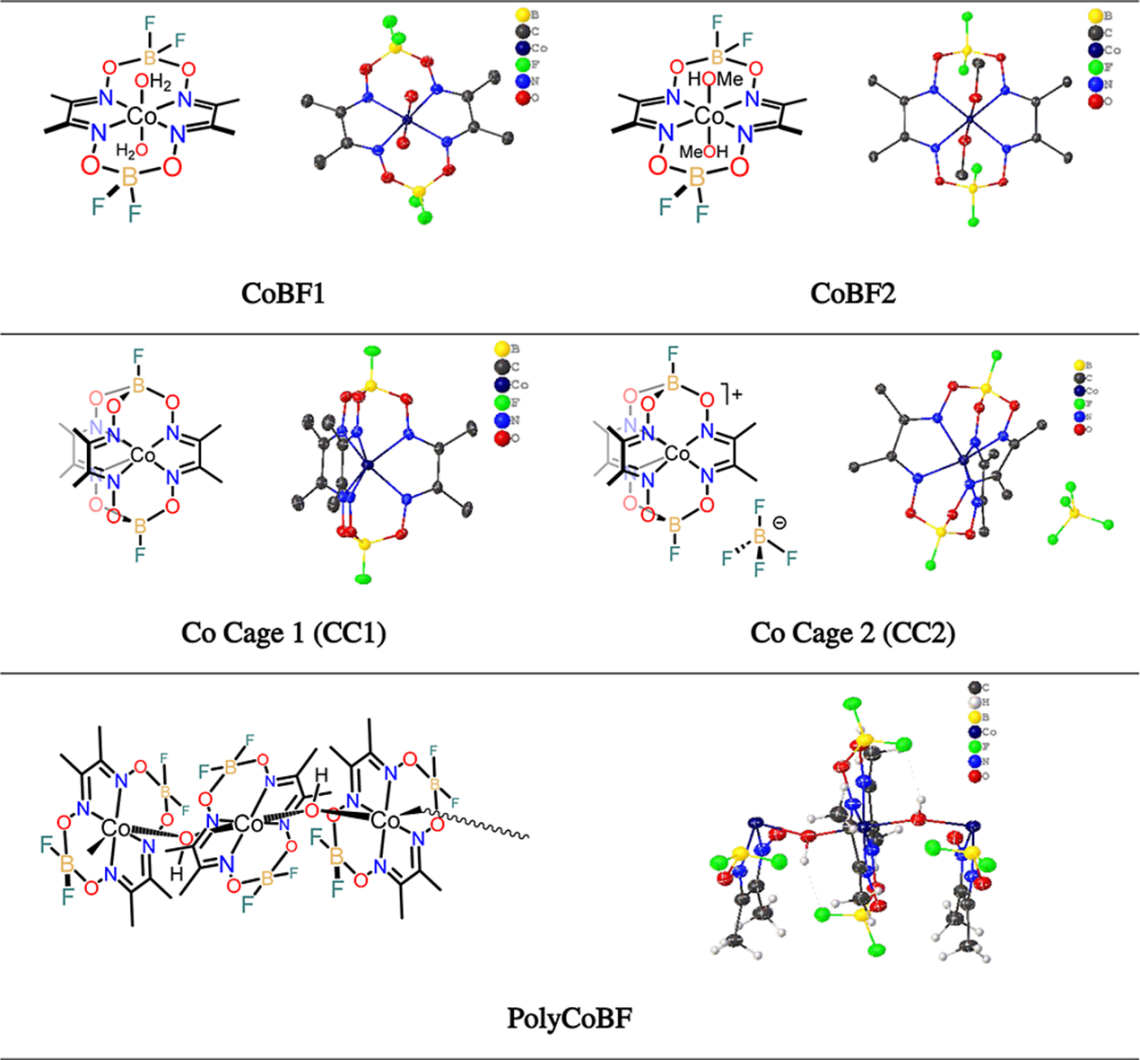
Phase structures identified in the CoBF samples.

The purpose of the crystallization was to determine the unknown structures of cobalt complexes synthesized using various methods. A given cobalt complex (10 mg) obtained via method 1(CoBF Ark) or 3 (CoBF method 3), was suspended in 2 mL of methanol or chloroform and subjected to ultrasonication for 15 min. It was then filtered through a membrane to obtain a clear solution, which was left in a fume hood to allow the solvent to evaporate completely at room temperature. The resulting crystals were suitable for single-crystal X-ray diffraction experiments and two new structures of CoBF1 (from the solution of CoBF method 3 in methanol) and PolyCoBF (from the solution of CoBF Ark in methanol) were discovered. These obtained crystals from both solutions were identified as a mixture of cobalt complexes, including CoBF2,^52^ cage1, cage2^53^ and the newly identified structures CoBF1 and PolyCoBF. The single-crystal structures are shown in Figure S1 and Figure 3.

### Bulk Thermal Polymerization of MMA

CoBF (1.92 mg, 5.0 × 10^–3^ mmol) was added to a 100 mL round-bottom flask, diluted with 50 g (53.2 mL, 0.5 mol) MMA. The mixture was sonicated and vortexed for 10 min to form a homogeneous stock solution of CoBF in MMA. A stock solution of AIBN in MMA was prepared 125 mg (0.76 mmol) of AIBN was added to a second 100 mL round-bottom flask with 50 g (53.2 mL, 0.5 mol) of MMA.

Six separate 50 mL Schlenk tubes with different amounts of CoBF stock solution and MMA with 5 g of AIBN stock solution were added, Table 1. The tubes were vortexed using small magnetic stirring bars for several minutes to form homogeneous solutions. Each Schlenk tube was subjected to 3 cycles of freeze–pump–thaw (∼3 × 10^–2^ mbar) on a Schlenk line to deoxygenate. The sealed Schlenk tubes were placed into a preheated oil bath at 70 °C and left to react for 10 min to obtain PMMA in relatively low conversions (∼5%). Afterward, the tubes were immediately cooled in an ice bath for 10 min to stop the reactions.

**Table 1:** Polymerization Reactions with Varying Amounts of Stock Solution and MMA, Total wt = 10 g

[CoBF]/[MMA] (ppm)	stock solution CoBF (g)	stock solution AIBN (g)	MMA (g)
0	0	5	5
1	1	5	4
2	2	5	3
3	3	5	2
4	4	5	1
5	5	5	0

### Gel Permeation Chromatography (GPC)

GPC analysis using THF as eluent was carried out using an Agilent 1260 Infinity II-MDS instrument equipped with a differential refractive index (DRI) detector, a variable wavelength UV/vis detector (VWD), a light scattering (LS) detector (15° and 90 ^o^) and a viscometer. The instrument was equipped with two PLgel Mixed C columns (300 × 7.5 mm), with a PLgel 5 μm guard column. The eluent used was THF with 0.01% w/v BHT (butylated hydroxytoluene) antioxidant. GPC samples were recorded at 1 mL/min at 30 °C. Agilent EasyVials PMMA standards over the range of 500–1.6 × 10^6^ g/mol were used for column calibration. The experimental molecular weight averages (*M*_n_, *M*_w_) and dispersity (*D̵*) of polymers were determined using Agilent GPC/SEC software.

### Nuclear Magnetic Resonance (NMR)

^1^H NMR (400 MHz) was recorded in CDCl_3_ (internal standard: 7.26 ppm) on a Bruker Avance III HD 400 MHz spectrometer. The chemical shifts were given in parts per million (ppm) and data was analyzed by ACD/NMR data software.

### Single Crystal X-ray Diffraction (SCXRD)

Suitable crystals were selected and mounted on a SuperNova, Dual, Cu at home/near, HyPix-6000HE diffractometer and a Rigaku Oxford Diffraction Synergy-S diffractometer with a dual source equipped with a HyPix-Arc 100-pixel hybrid photon counting detector. During data collection, the crystal was kept at a steady *T* = 100.02(18) K. The structure was solved with the *ShelXT*(54) solution program using the intrinsic phasing method and by using *Olex*2 1.5^55^ as the graphical interface. The model was refined with *ShelXL* 2019/3^54^ using full matrix least-squares minimization on *F*^2^.

### Powder X-ray Diffraction (PXRD)

**Figure 4:**
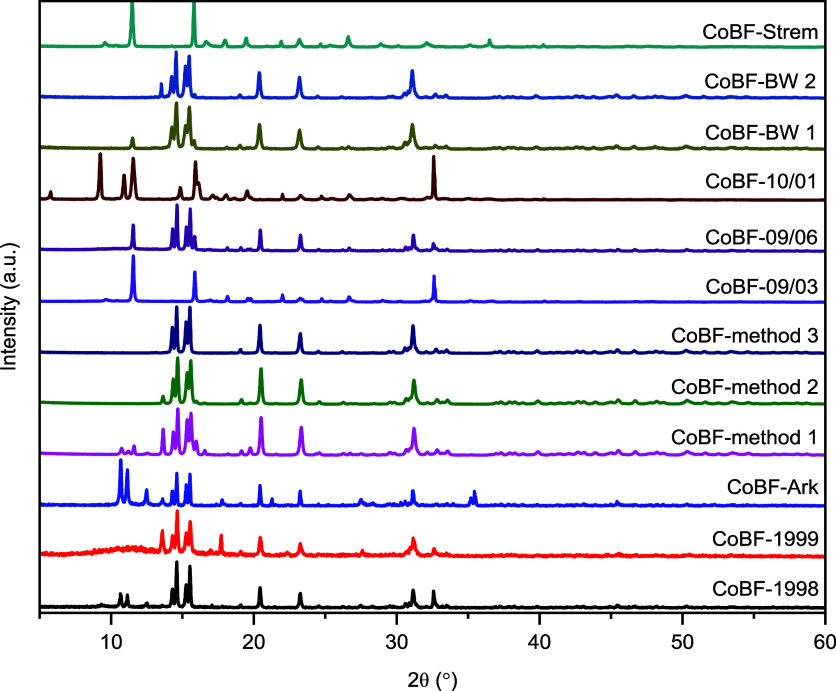
Powder XRD patterns of the 12 CoBF samples.

XRD measurements were performed on Anton Paar powder X-ray diffractometer (XRDynamic500) using Co-*K*α radiation (λ = 1.78901 Å) and the diffraction patterns covering the range of 2θ from 5° to 60°. The diffraction data were collected with a step size of 0.01° and a scan time of 80 s per step. The data were analyzed using the software *Profex* 5.3.1.^56^ The patterns of the 12 CoBF samples are shown in Figure 4 and analysis results are displayed in Section S2 in Supporting Information.

### Fourier Transform Infrared Spectroscopy (FTIR)

FTIR was recorded on a Bruker Alpha II spectrometer with a detection range from 500 to 4000 cm^–1^.

### X-ray Photoelectron Spectroscopy (XPS)

The samples investigated in this study were attached to electrically conductive carbon tape and mounted onto a sample bar with a layer of filter paper between the samples and the sample bar to ensure electrical isolation and hence differential charging, before being loaded into a Kratos Axis Ultra DLD spectrometer which possesses a base pressure below 1 × 10^–10^ mbar. XPS measurements were performed in the main analysis chamber, with the sample being illuminated using a monochromated Al *K*_α_ X-ray source (*h*ν = 1486.7 *e*V). The measurements were conducted at room temperature and at a takeoff angle of 90° with respect to the surface parallel. The core level spectra were recorded using a pass energy of 20 eV (resolution approximately 0.4 eV), from an analysis area of 300 mm × 700 mm. The work function and binding energy scale of the spectrometer were calibrated using the Fermi edge and 3d_5/2_ peak recorded from a polycrystalline Ag sample prior to the commencement of the experiments. To prevent surface charging the surface was flooded with a beam of low-energy electrons from a charge neutralizer throughout the experiment and this necessitated recalibration of the binding energy scale. To achieve this, the C–C/C–H component of the C 1s spectrum was referenced to 285.0 eV. The data were analyzed in the *CasaXPS* package using Shirley backgrounds and mixed Gaussian–Lorentzian (*Voigt*) line shapes. For compositional analysis, the analyzer transmission function has been determined using clean metallic foils to determine the detection efficiency across the full binding energy range.^57,58^

## Results and Discussion

Figure 3 illustrates the chemical structures for some phases identified in the powders and the single crystal structures obtained after the crystallization experiments. The two cobalt(II) complexes CoBF1 and CoBF2 (CCDC Refcode FAPRET) are the effective catalysts and the structure of CoBF2 has previously been reported in 1986.^52^ The single crystal structure of CoBF1 (Figure 3) is a new structure, and single crystals were obtained from chloroform solution in this work. The structures of CoBF1 and CoBF2 show that Co(II) is directly bound to water and methanol, vibration peaks appear at 3526–3596 cm^–1^ in the FTIR spectrum (Figure S50).

A new interesting crystal structure (PolyCoBF) was also discovered from the crystal growth experiment where a methanol solution was used, Figure 3b. This structure shows cobalt(III) bridged by OH groups leading to an unusual co-ordination polymer. The ligand units of the O–BF_2_–O moiety in this complex orient above and below the main equatorial plane, adopting an extended chair conformation.^59^ We decided to try and use XPS to help confirm the oxidation state and to distinguish between Co(II) and Co(III). We recorded XPS of cobalt(II) chloride hexahydrate, cobalt(III) acetylacetonate and Chloro(pyridine)bis(dimethylglyoximato)cobalt(III) as reference samples (Figures S5.1–S5.3). By fitting the Co 2p_3/2_ region, the 3p_1/2_ from the spin–orbit splitting can be seen in the spectra. For typical Co(II) we observed a main peak at ∼782 eV (Figure S51), with large adjacent satellite features. However, PolyCoBF (Figure S54) appeared very similar to the Co(III) structures (Figure S52, S53) suggesting a Co^3+^ oxidation state, further supporting that the −OH–Co–OH– group in the polymeric structure (Section S5 in Supporting Information).

One anhydrite caged structure CC1 (CCDC Refcode COXAME) and a second hydrate CC2 (CCDC Refcode OAMECO) (Figure 3) were obtained from the same crystallization experiment from methanol, as previously reported by Lingafelter in 1971.^53^

The X-ray diffraction patterns of the 12 CoBF samples are shown in Figure 4. The peaks located at approximately the 2θ position = 15°, 23° are present in every sample, suggesting partial similarity in the composition, however, significant differences are observed for the peaks in the 2θ range (5–60°) which vary from sample to sample. Thus, the observed phases are informative of practical significance helping optimization of the CoBF synthesis route and as a measure of stability and purity.

**Figure 5:**
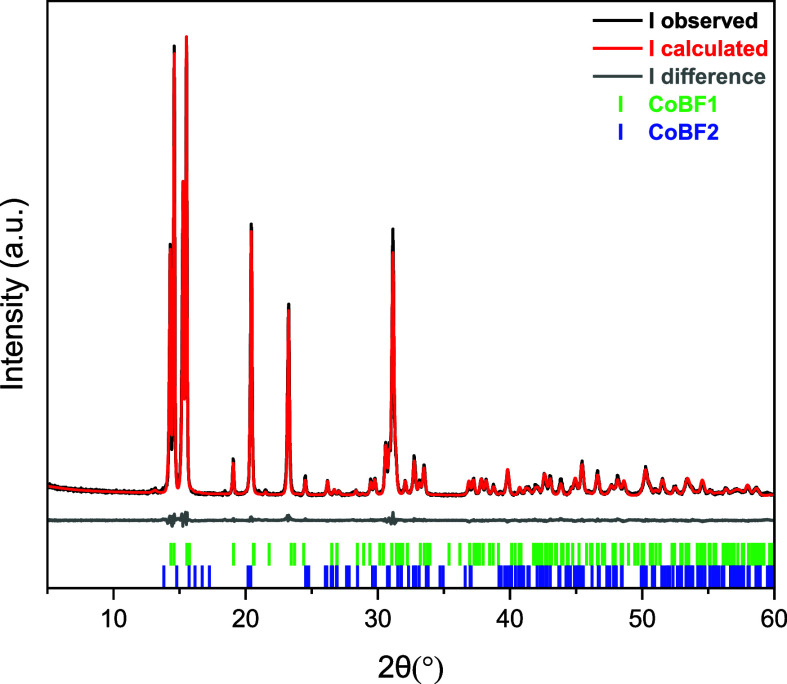
Refinement of one CoBF sample (CoBF-method 3). The experimental pattern is in black, the calculated pattern is in red, the difference between the experimental and calculated pattern is in gray, and the vertical lines refer to the different simulated powder X-ray patterns of each crystallite calculated from the SCXRD data.

Eleven of the CoBF samples used in the study were synthesized and one was commercially obtained. The powder XRD was collected for all 12 CoBF samples by the same diffractometric method. One example of XRD refinement data is shown in Figure 5 for illustrative purposes and the others are all reported in Section S2 (Supporting Information).

The XRD pattern of the CoBF sample synthesized via method 3 is shown in Figures 5 and S7. The crystalline phases were identified and quantified using the PXRD patterns simulated from SCXRD data of the obtained structures as well as the CCDC database.

The estimation of the content of each crystalline form in the bulk sample was performed from the intensity integration of the areas in the computationally calculated diffraction pattern. The curves from the experimental spectrum and the simulated data showed good agreement (*R*_wp_ = 10.62%, GOF = 1.4048). The analysis of the XRD data showed the sample with the following phases with a relative weight percentage of active catalyst form (Table 2): CoBF1 (98.3%), CoBF2 (1.7%).

**Table 2:** Constituent Data Fit of Sample CoBF-Method 3 as Calculated from Figure 5

parameter	value (wt %)	ESD
CoBF1	98.3	0.001
CoBF2	1.7	0.001

The phase identification and quantitative analysis results for all samples performed using the Rietveld refinement method with *Profex* 5.3.1 software^56^ are shown in Figures S2–S13 (Section S2 in the Supporting Information.). The structures of the cobalt complexes (CoBF1, CoBF2, PolyCoBF, Co Cage1 (marked as Cage1 in the legend), and Co Cage2 (marked as Cage2 in the legend), along with the raw material dimethylglyoxime (DMG) and NaHCO_3_ (present after purification), were used as reference phases for the refinement. Other phases were not considered in this study as the unidentified peaks (marked with ★ in the XRD patterns) corresponded to unknown phases which could not be obtained despite multiple crystallization attempts. The weight percentage of components for the 12 CoBF samples is listed in Table 3. The composition analysis results reveal the unreacted component dimethylglyoxime or Co Cage (CC1 and CC2), which was assumed due to the nonstochiometric amounts of dimethyl glyoxime used in CoBF synthesis. The component NaHCO_3_ is present from residual amounts used during the neutralization of the HF step. The amorphous impurities could be formed following the boron trifluoride diethyl etherate reaction with cobaloxime. Notably, for sample CoBF 10/01, CoBF 09/03, and Strem CoBF, the predominant phase is Cage2, alongside other unidentified impurities. The amorphous and unidentified phase amounts are reported in Table 3. For PolyCoBF (indicated in cyan lines in Figures S2–S6, S9, S10), the first 2θ peak in the calculated PXRD pattern appears at 7.673°(marked with an arrow in Figure S2b). However, the intensity of this peak is significantly weaker than the peaks in the 10–12° range (Figure S2b). This peak is not visible in the recorded diffraction patterns of samples CoBF 1998 (Figure S2), CoBF 1999 (Figure S3), and CoBF Method 1 (Figure S5), whereas the peaks around 10–12° are clearly observed. The preferred orientation effects may contribute to the reduced intensity or disappearance of this peak.

**Table 3:** Component Content Data of the CoBF Powder Samples as Calculated by Powder XRD

catalyst sample name	component content (wt %)							
	CoBF1	CoBF2	Poly CoBF	CC1	CC2	DMG	NaHCO_3_	amorphous/unidentified
CoBF 1998	73.2	3.7	11.0			2.2	0.9	9.1
CoBF 1999	43.6	3.0	8.6	9.8	0.9			34.1
CoBF Ark	47.7		34.7	6.3				11.3
CoBF method 1	79.9	9.9	3.9	1.2	5.1			
CoBF method 2	91.8	6.4	1.8					
CoBF method 3	98.3	1.7						
CoBF09/03		6.5			84.8			8.7
CoBF09/06	68.6	15.4	1.2		14.8			
CoBF10/01		5.5	2.1		60.8	11.3		20.3
BW CoBF 1	91.9	2.1			6.0			
BW CoBF 2	88.4	8.8						2.8
strem CoBF	13.3	0.3			83.6			2.8

**Figure 6:**
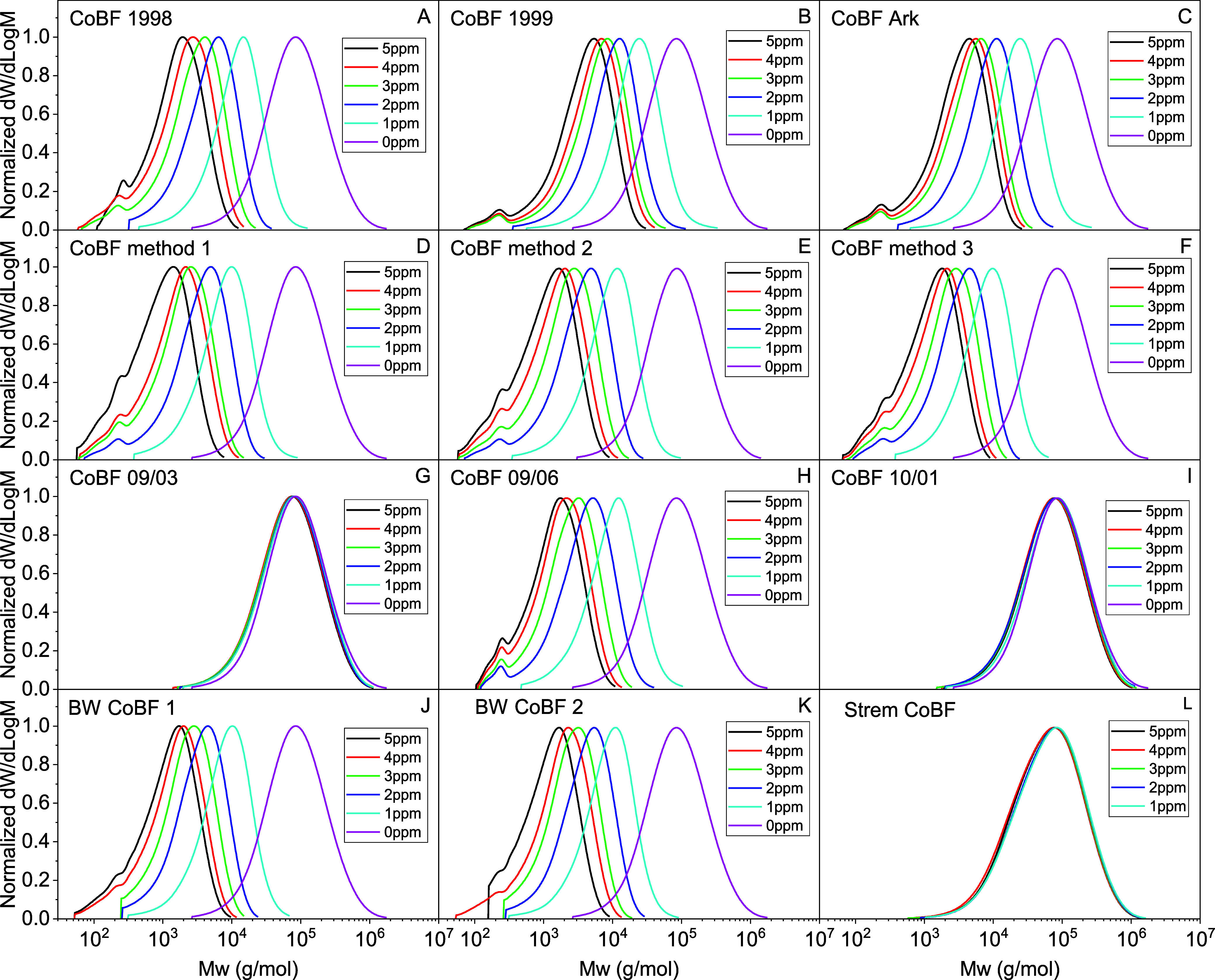
GPC of the bulk polymerization of MMA using the 12 different CoBF samples with [CoBF] between 0 and 5 ppm.

CCTP of MMA with the 12 samples of CoBF was carried out using the bulk thermal polymerization method, and the GPC results, Figure 6.

In the experiments, the [CoBF]/[MMA] was changed, as shown in Table 1, the amount of initiator was kept constant, and the temperature was kept at 70 °C. As shown in Figure 6 and Table 4, a reduction in molecular weight would be expected with an increase in the concentration of CoBF. The Weight-average Molecular Weight (*M*_w_) of the polymer changed slightly from 1.3 × 10^5^ g/mol to 1.1 × 10^5^ g/mol with the amount of CoBF from 0 to 5 ppm for CoBF 09/03, CoBF 10/01 and Strem CoBF. Whereas the molecular weight range varied from 1.3 × 10^5^ g/mol to 10^3^ g/mol for the other 9 CoBF samples. The *DP*_w_/2 (derived from *M*_w_) was used instead of *DP*_n_ to avoid errors associated with changes in dispersity especially due to the low molecular weight components.^60−62^

**Table 4:** Different CoBF Samples with Various Amounts of CoBF Were Used in Experiments and Calculated *C*_s_ Values

catalyst sample	weight-average molecular weight (*M*_w_) with different [CoBF]/[MMA] (g mol^–1^) *C*_s_						
	0 ppm	1 ppm	2 ppm	3 ppm	4 ppm	5 ppm	
CoBF 1998	130,000	16,000	6500	3900	2700	2100	19,300
CoBF 1999	130,000	30,000	13,300	8500	6900	5100	7500
CoBF Ark	130,000	29,300	11,500	6400	5300	4200	9700
CoBF method 1	130,000	10,800	4800	2600	2100	1300	24,700
CoBF method 2	130,000	12,300	4700	2800	1900	1500	27,400
CoBF method 3	130,000	10,100	4300	2900	2000	1600	24,800
CoBF09/03	130,000	117,900	113,200	113,700	109,800	109,200	33
CoBF09/06	130,000	13,700	5500	3300	2300	1900	22,000
CoBF10/01	130,000	122,200	116,900	114,300	109,900	109,700	48
BW CoBF 1	130,000	10,800	4500	3000	2000	1600	25,200
BW CoBF 2	130,000	11,600	5500	3300	2400	1600	23,900
Strem CoBF	130,000	114,800	112,600	110,400	104,900	106,300	35

**Figure 7:**
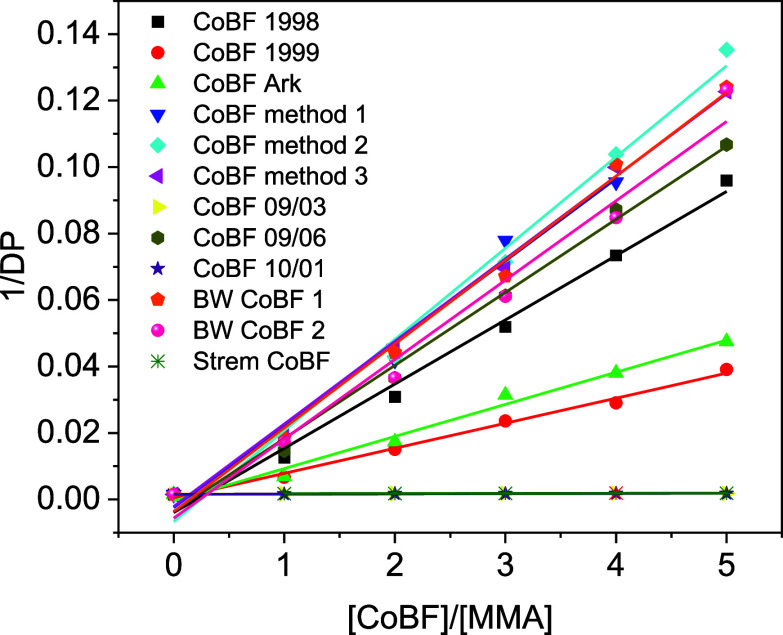
Mayo plots of CoBF 1998, CoBF 1999, CoBF Ark, CoBF method 1, CoBF method 2, CoBF method 3, CoBF 09/03, CoBF 09/06, CoBF 10/01, BW CoBF 1, BW CoBF 2 and Strem CoBF.

Figure 7 is plotted using the data in Table 4, illustrating the relationship between catalyst activity and chain transfer efficiency. Samples with a lower slope (close to 0) in the fit line (CoBF 09/03, CoBF 10/01, and Strem CoBF) exhibit smaller catalyst activity, whereas a steeper slope indicates enhanced chain transfer efficiency for the rest samples.

The composition of CoBF obtained by the Rietveld refinement of the XRD patterns is summarized in Table 5,^56^ demonstrating that the CoBF samples with three different methods (1, 2 and 3) and the samples BW COBF (1 and 2) had high *C*_s_ values ranging from 23,900 to 27,400. The significant decrease in catalytic efficiency of previously made CoBF samples (1998, 1999 and Ark) may be due to poor storage conditions. Surprisingly, the commercial sample (Strem CoBF) showed a very high level of impurity (86.4%) with *C*_s_ value = 35 comparable to the sample CoBF 09/03 and CoBF 10/01. There was less PolyCoBF present in the sample from method 3 than method 1, however, 34.7% of PolyCoBF was observed in the sample CoBF Ark.

**Table 5:** Composition and *C*_s_ Value of Each of the CoBF Samples^a^

	CoBF (%)	PolyCoBF (%)	impurity (%)	chain transfer constant *C*_s_
CoBF 1998	76.9	11.0	12.2	19,300
CoBF 1999	46.6	8.6	44.8	7500
CoBF Ark	47.7	34.7	17.6	9700
CoBF method 1	89.8	3.9	6.3	24,700
CoBF method 2	98.2	1.8	<0.01	27,400
CoBF method 3	100.0	<0.01	<0.01	24,800
CoBF09/03	6.5	<0.01	93.5	33
CoBF09/06	84.0	1.2	14.8	22,000
CoBF10/01	5.5	2.1	92.4	48
BW CoBF 1	94	<0.01	6	25,200
BW CoBF 2	97.2	<0.01	2.8	23,900
Strem CoBF	13.6	<0.01	86.4	35

a The PolyCoBF is the amount of only itself, the solubility (at RT) of PolyCoBF is minimal in MMA and thus was not measured, the effect of PolyCoBF on the CCTP was ignored in this analysis. The impurity contains all the caged structures of cobalt complexes, unreacted raw materials, amorphous and unidentified solids which do not participate in CCTP.

**Figure 8:**
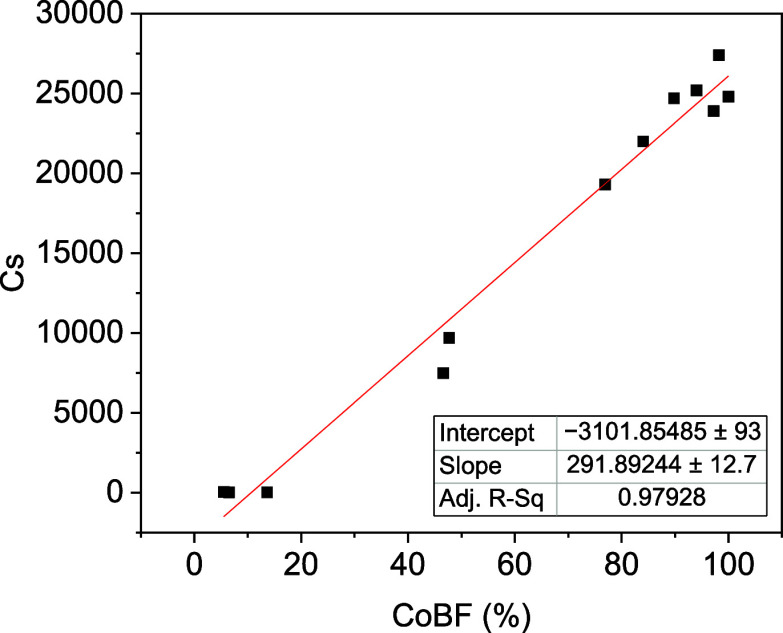
Experimental *C*_s_ values were calculated from Mayo plots versus the constituent of CoBF (crystalline CoBF1 and CoBF2) in powder samples.

To investigate the relationship between the proportion of active CoBF (or catalyst) in each of the samples as determined from refined and calculated from XRD and the catalytic chain transfer coefficient from bulk polymerization of MMA, the *C*_s_ value vs effective CoBF content (the constituents of crystalline CoBF1 and CoBF2) plotted. The measured *C*_s_ value is directly proportional to the effective constituent of CoBF, Figure 8, which means the purer the CoBF sample is, the higher the efficiency of the CCTP reaction becomes. Thus, for any CoBF sample with unknown composition, the quantitative analysis of PXRD patterns can be an effective way to estimate the *C*_s_ value, and catalyst activity without any time-consuming polymerization reaction being required.

## Conclusions

We have been able to identify several cobalt complexes present in the synthesized CoBF which change depending on the synthetic method and age of the sample. Determination of the crystal structures of isolated crystals allows for the resolution of powder patterns for each species. Analysis of catalyst samples which contain complex mixtures by powder XRD then allows for the composition of the mixtures to be determined. The CCTP activity of each species has been determined which then in turn allows the CCTP activity to be predicted from the knowledge of the composition of the catalyst sample from XRD. This predicted activity has been shown to have an excellent correlation. To test this method samples from two different laboratories were sourced which had been synthesized and, in some cases, stored over many years and the powder XRD allowed us to determine the composition and predict activity with good accuracy and confidence. We also noticed that when the catalyst is stored over many years the activity reduces, and this also correlates directly with the CCTP activity. CCTP has been used commercially for over 35 years by multiple companies and academic laboratories without a publicly reported high-yielding synthesis or method of determining catalyst purity/activity other than carrying out polymerization reactions we report a relatively simple method that allows this to be determined as well as reporting the structure of some new cobalt compounds found. We note that surprisingly the least active and least pure catalyst was the commercial sample, underpinning the importance of this current work. This XRD method gives a quantitative method that complements the more qualitative UV/vis method reported by one of the authors recently.^46^

## Supporting Information Available

Including XRD patterns and refinement results, NMR, XPS, IR spectrum, and bulk polymerization results (PDF)

## Abbreviations

CCTP: catalytic chain transfer polymerization
CoBF, MMA: methyl methacrylate
PMMA: poly(methyl methacrylate)
AIBN: 2,2′-azobis(2-methylpropionitrile)
PXRD: powder X-ray diffraction
SCXRD: single crystal X-ray diffraction.
