# Supplementary material for: Assessing Cobalt(II/III) Complex Purity Using XRD and Its Impact on Effectiveness of Catalytic Chain Transfer Polymerization
Source: Macromolecules. 2025 Mar 6;58(6):3188–98. doi: 10.1021/acs.macromol.5c00049 (PMC11948481; doi:10.1021/acs.macromol.5c00049)
Supplement: Supplementary file 1 — ma5c00049_si_001.pdf [file ma5c00049_si_001.pdf]

Supporting information

Assessing Cobalt (II/III) Complex Purity Using XRD and Its Impact on Effectiveness of Catalytic Chain Transfer Polymerization

Xiaofan Yang[a], Jie Liu[b], Nicholas R. Bagnall[d], Johan P.A. Heuts[c], Brady Worrell[d], Christopher Waldron[e], David M Haddleton*[a]*

[a] *Department of Chemistry, University of Warwick, Coventry, CV4 7AL*

[b] *Department of Physics, University of Warwick, Coventry, CV4 7AL*

[c] *Department of Chemical Engineering & Chemistry and Institute for Complex Molecular Systems, Eindhoven University of Technology, PO Box 513, 5600 MB Eindhoven, The Netherlands*

[d] *University of Denver, Department of Chemistry & Biochemistry, Denver, CO 80210 USA*

[e] Research and Technology Platforms, University of Warwick, Coventry, CV4 7AL

**d.m.haddleton@warwick.ac.uk*

Contents

1 SCXRD data of Cobalt complexes 3

2. Analysis of the PXRD data of CoBF samples 7
2.1 CoBF 1998 7
2.2 CoBF 1999 9
2.3 CoBF Ark 10
2.4 CoBF method 1 11
2.5 CoBF method 2 12
2.6 CoBF method 3 13
2.7 CoBF 09/03 14
2.8 CoBF 09/06 15
2.9 CoBF 10/01 16
2.10 BW CoBF 1 17
2.11 BW CoBF 2 18
2.12 Strem CoBF 19
3 Bulk polymerization of MMA with different CoBF samples 20
3.1 CoBF 1998 20
3.2 CoBF 1999 22
3.3 CoBF Ark 24
3.4 CoBF method 1 26
3.5 CoBF method 2 28
3.6 CoBF method 3 30
3.7 CoBF 09/03 32
3.8 CoBF 09/06 34
3.9 CoBF 10/01 36
3.10 BW CoBF 1 38
3.11 BW CoBF 2 40
3.12 Strem CoBF 42
4. Infrared spectrum 44
5. X-ray photoelectron spectroscopy 45
5.1 Cobalt(II) chloride hexahydrate 45
5.2 Cobalt(III) acetylacetonate 45
5.3 Chloro(pyridine)bis(dimethylglyoximato)cobalt(III) 46
5.4 PolyCoBF 46
References 47

1 SCXRD data of Cobalt complexes

Table S1. Crystallographic data and refinement details.

Name		CoBF1	CoBF2[a]	PolyCoBF	Co Cage 1[b]	Co Cage 2[b]
Formula		$C_8H_{16}B_2CoF_4N_4O_6$	$C_{10}H_{22}B_2CoF_4N_4O_6$	$C_{16}H_{26}B_4Co_2F_8N_8O_{10}$	$C_{12}H_{18}B_2CoF_2N_6O_6$	C12 H18 B3 Co F6 N6 O6
Temperature/K		100.15(2)	100.0(2)	100.0(2)	293(10)	293(10)
Crystal System		triclinic	triclinic	triclinic	Trigonal	orthorhombic
Space group		P-1	P-1	P-1	R-3c	Fdd2
Unite cell dimensions	*a*/Å	6.7549(4)	7.8683(3)	9.7363(2)	11.543(1)	16.582(1)
	b/ Å	7.8496(7)	7.9527(5)	11.3243(4)	11.543(1)	20.844(3)
	c/ Å	7.8786(6)	8.1271(4)	14.2504(5)	22.849(5)	12.191(1)
	α/°	66.026(8)	66.290(5)	71.660(3)	90	90
	β/°	78.624(6)	79.613(4)	77.503(3)	90	90
	λ/°	88.135(6)	62.080(5)	71.647(3)	120	90
Volume		373.68(5)	411.40(4)	1403.48(8)	2636.55	4213.6(7)
Z		1	1	2	6	8
I/*σ* (I) (max resltn)		43.1	56.8	51.8	/	/
Data Completeness		100	99.6	99.1	/	/
Goodness-of-fit on F^2		1.045	1.091	1.151	/	/
Final *R* indices [I>2 σ (I)]	R_1	0.0340	0.0295	0.0766	0.043	0.061
	wR_2	0.0891	0.0793	0.2169	0.078	0.033
R indices (all data)	R_1	0.0341	0.0295	0.0811	/	/
	wR_2	0.0893	0.0793	0.2211	/	/

$R_1 = \Sigma \|\|Fo\|-\|Fc\|\| / \Sigma \|Fo\|$, $wR_2 = \{\Sigma [w(Fo^2 - Fc^2)^2] / \Sigma [w(Fo^2)^2]\}^{1/2}$

[a] *Parameters of CoBF2 are refined from the single crystals obtained through recrystallization in this work.*
[b] *Parameters of Co Cage1 and Co cage2 structures are from the CCDC database with Refcode* **COXAME** *and* **OAMECO**.[1]

Table S2. The bond length of Co-O and Co-N

Name	CoBF1	CoBF2	PolyCoBF	Co Cage 1
Co-O1	2.2751(17)	2.2508(14)	1.942(5)	/
Co-O2	/	/	1.949(5)	/
Co-O3	/	/	1.959(5)	/
Co-O4	/	/	1.962(5)	/
Co-N1	1.8874(19)	1.8796(17)	1.880(5)	1.97(3)
Co-N2	1.8816(18)	1.8834(17)	1.873(5)	/
Co-N3	/	/	1.884(5)	/
Co-N4	/	/	1.868(5)	/
Co-N5	/	/	1.877(5)	/
Co-N6	/	/	1.876(5)	/

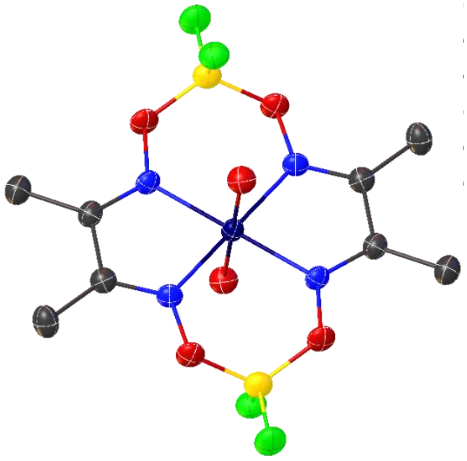

CoBF 1

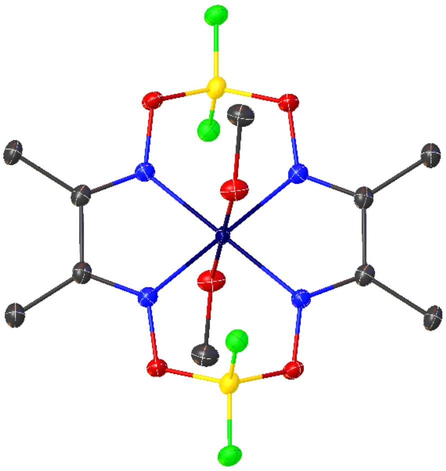

CoBF 2

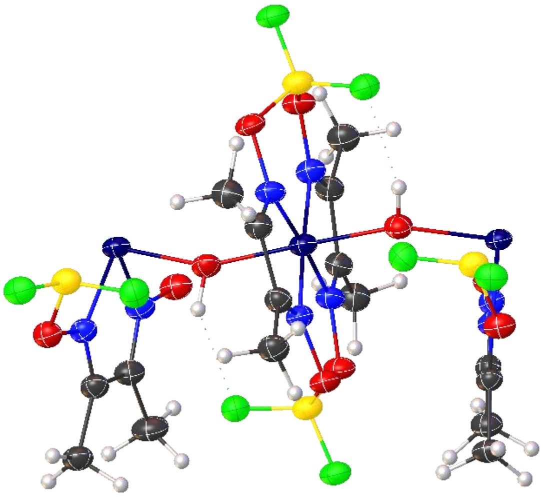

PolyCoBF

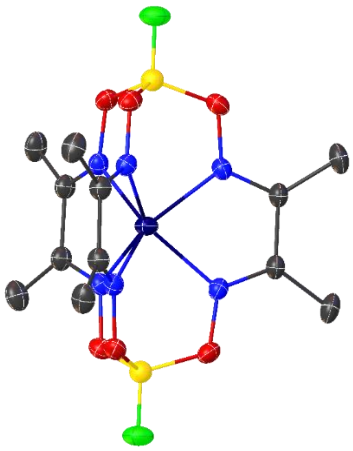

Co Cage 1

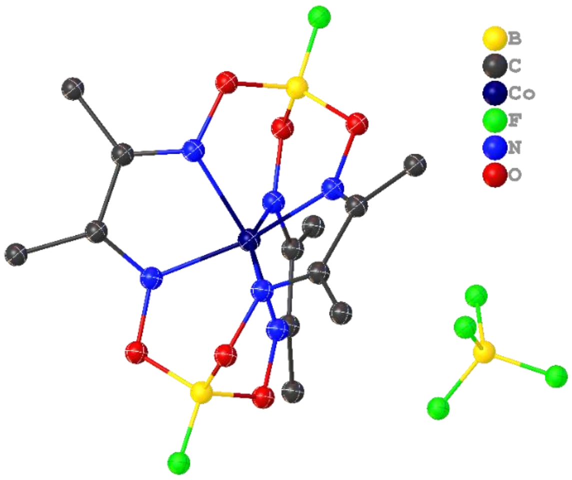

Co Cage 2

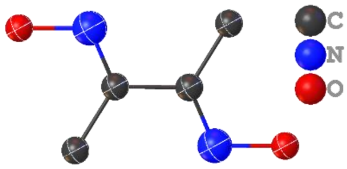

Dimethylglyoxime[c]

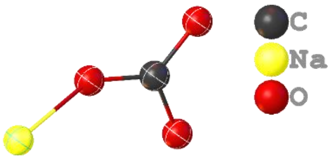

NaHCO₃[c]

Figure S1. *Crystal structures of raw materials and products*

[c]*Single crystal structures of Dimethylglyoxime and $NaHCO_3$ are from CCDC database with the Refcode* **DMEGLY05** *and* **UDOREM05**, *respectively.*

2. Analysis of the PXRD data of CoBF samples

The quantitative composition analysis of the 12 **CoBF** *samples was performed using the Rietveld refinement method*[1] *using software Profex 5.3.1.*[2] *The structures of the cobalt complexes (***CoBF1, CoBF2, PolyCoBF, Co Cage1, and Co Cage2***), along with the raw material dimethylglyoxime (***DMG***) and* **$NaHCO_3$** *(exceeded after purification), were used as reference phases for the refinement. Other phases were not considered in this study as the unidentified peaks corresponded to unknown phases. Additionally, single crystals for the unknown compounds could not be obtained despite multiple crystallisation attempts. Regarding this, the unidentified peaks (marked with ★) correspond to unknown phases for which crystallographic information could not be determined from our crystallisation experiments. For the samples containing amorphous and unidentified phases, the percentage of the component and the amorphous and unidentified phases are calculated below:*

1. *The percentage of the crystalline component is calculated as: the refined component percentage × total area of all crystalline peaks / (total area of all crystalline peaks + amorphous and unidentified peaks).*
2. *The percentage of amorphous and unidentified phases is calculated as: the total area of all amorphous and unidentified peaks / (total area of all crystalline peaks+ amorphous and unidentified peaks).*

Notably, for samples CoBF 09/03, CoBF 10/01, and Strem CoBF, the predominant phase is Cage2, which doesn't have catalytic effectiveness. The presence of an unidentified phase in these samples affected the refinement results. Consequently, the component compositions were recalculated based on the amount of the amorphous and unidentified phases using the method 1 and 2 described above.

2.1 CoBF 1998

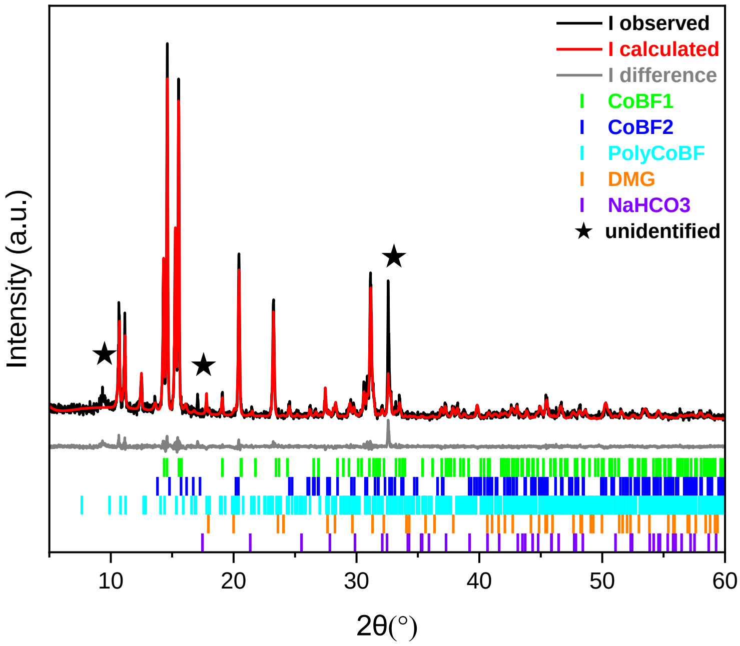

Figure S2a. *The refinement result for CoBF 1998 sample. The experimental data is in black, and the calculated pattern is in red. ★ representing unidentified peak associates to unknown phases. R_{wp}=27.91%, GoF=1.2225. The vertical lines in different colours under the XRD pattern represent CoBF1 (in green), CoBF2 (in blue), PolyCoBF (in cyan), DMG (in orange), $NaHCO_3$ (in purple).*

Table S3. *Composition of the Sample CoBF 1998 obtained from the above refinement.*

Parameter	*Value*	*ESD*
CoBF1	*0.732*	*0.05*
CoBF2	*0.0366*	*0.04*
PolyCoBF	*0.1098*	*0.03*
DMG	*0.0217*	*0.02*
$NaHCO_3$	*0.0091*	*0.03*
Amorphous and unidentified(★)[a]	*0.0908*	*/*

[a] *: The unidentified peaks (marked with ★) correspond to unknown phases for which crystallographic information could not be determined from our crystallisation experiments.*

Amorphous and unidentified %= total area of all Amorphous and unidentified peaks /total area of all crystalline and amorphous and unidentified peaks.

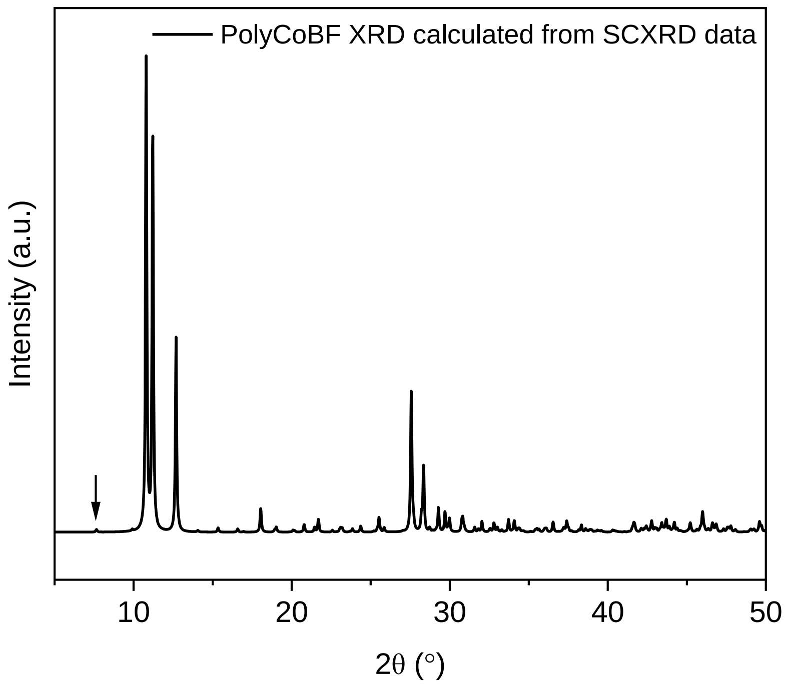

Figure S2b. *The XRD pattern of the PolyCoBF calculated from the SCXRD data. The first peak at 2θ=7.673° was marked with an arrow.*

2.2 CoBF 1999

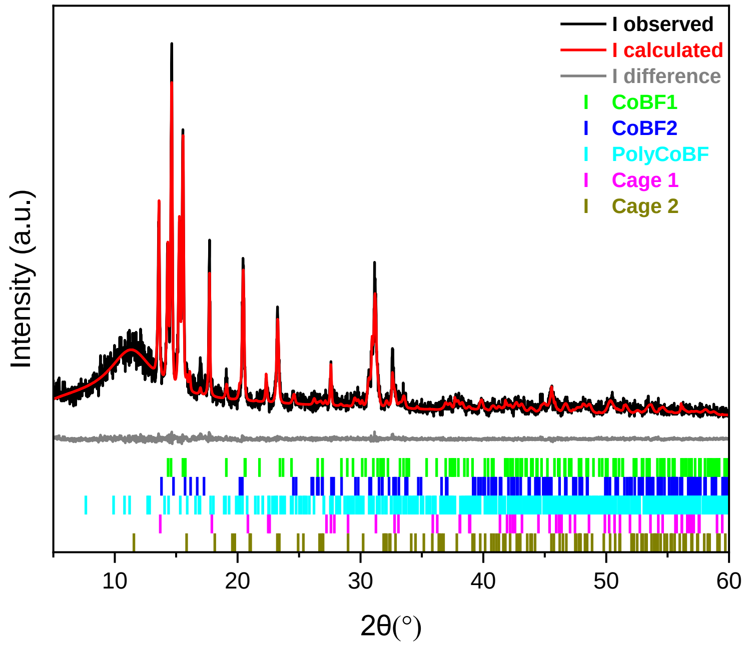

Figure S3. *The refinement result for CoBF 1999 sample. The experimental data is in black, and the calculated pattern is in red. R_{wp}=21.03%, GoF=1.1133. The vertical lines in different colours under the XRD pattern represent CoBF1 (in green), CoBF2 (in blue), PolyCoBF (in cyan), Cage 1 (in pink), and Cage 2(in olive).*

Table S4. *Composition of the Sample CoBF 1999 obtained from the above refinement.*

Parameter	*Value*	*ESD*
CoBF1	*0.4356*	*0.02*
CoBF2	*0.03036*	*0.008*
PolyCoBF	*0.0858*	*0.03*
Co Cage 1	*0.09834*	*0.007*
Co Cage 2	*0.00924*	*0.004*
Amorphous [a]	*34.1*	*/*

[a] *: Amorphous %= total area of all Amorphous peaks /total area of all crystalline and amorphous peaks.*

2.3 CoBF Ark

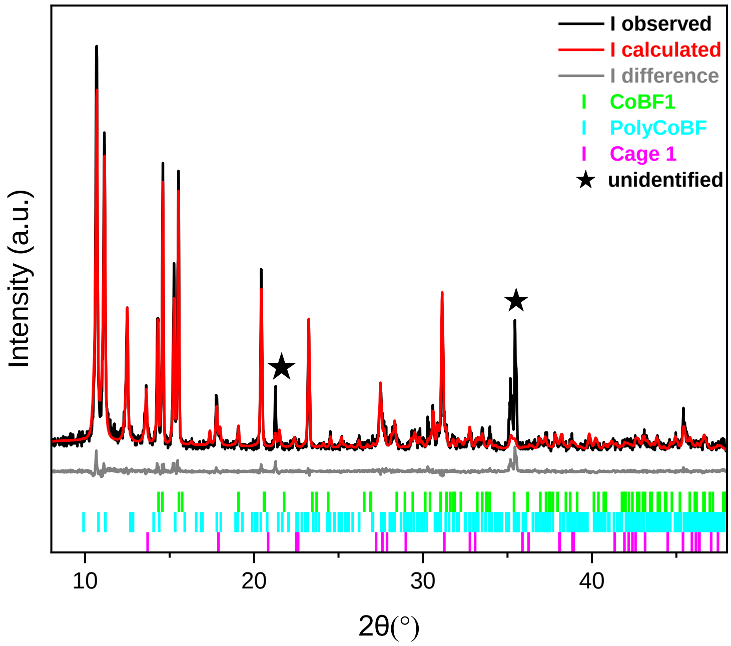

Figure S4. *The refinement result for CoBF Ark sample. The experimental data is in black, and the calculated pattern is in red. The peaks with★mark are from unidentified materials. R_{wp}=35.11%, GoF=1.3823. The vertical lines in different colours under the XRD pattern represent CoBF1 (in green), PolyCoBF (in cyan), Cage 1 (in pink).*

Table S5. *Composition of the Sample CoBF Ark obtained from the above refinement. The peaks with★mark are calculated in the amorphous and unidentified part.*

Parameter	*Value*	*ESD*

CoBF1	*0.47702*	*0.006*
PolyCoBF	*0.34733*	*0.005*
Co Cage 1	*0.06307*	*0.004*
Amorphous and unidentified(★)[a]	*0.11258*	*/*

[a] : The unidentified peaks (marked with ★) correspond to unknown phases for which crystallographic information could not be determined from our crystallisation experiments.
Amorphous and unidentified %= total area of all Amorphous and unidentified peaks /total area of all crystalline and amorphous and unidentified peaks.

2.4 CoBF method 1

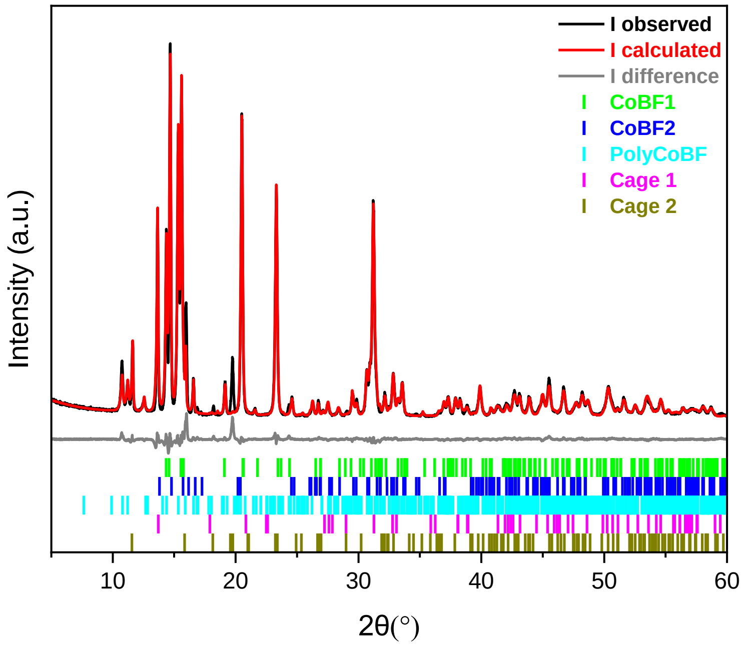

Figure S5. *The refinement result for CoBF method 1 sample. The experimental data is in black, and the calculated pattern is in red. R_{wp}=8.85%, GoF=2.1223. The vertical lines in different colours under the XRD pattern represent CoBF1 (in green), CoBF2 (in blue), PolyCoBF (in cyan), Cage 1 (in pink), Cage 2(in olive)*

Table S6. *Composition of the Sample CoBF method 1 obtained from the above refinement.*

Parameter	*Value*	*ESD*
CoBF1	*0.799*	*0.002*
CoBF2	*0.0986*	*0.001*
PolyCoBF	*0.0392*	*0.0004*
Co Cage 1	*0.0120*	*0.002*
Co Cage 2	*0.0510*	*0.001*

2.5 CoBF method 2

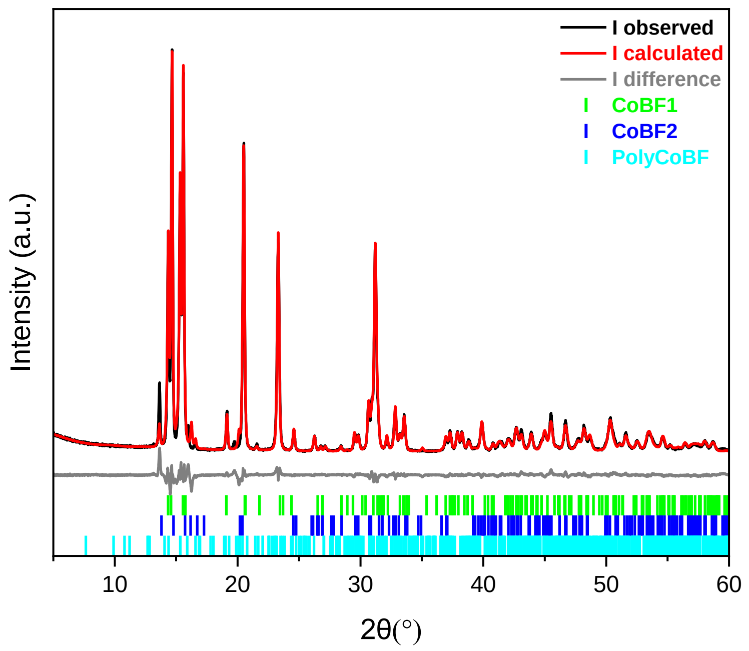

Figure S6. *Refinement result for CoBF method 2 sample. The experimental data is in black, and the calculated pattern is in red. R_{wp}=10.16%, GoF=2.4019. The vertical lines in different colours under the XRD pattern represent CoBF1 (in green), CoBF2 (in blue), PolyCoBF (in cyan).*

Table S7. *Composition of the Sample CoBF method 2 obtained from the above refinement.*

Parameter	*Value*	*ESD*
CoBF1	*0.918*	*0.003*
CoBF2	*0.064*	*0.001*
PolyCoBF	*0.018*	*0.002*

2.6 CoBF method 3

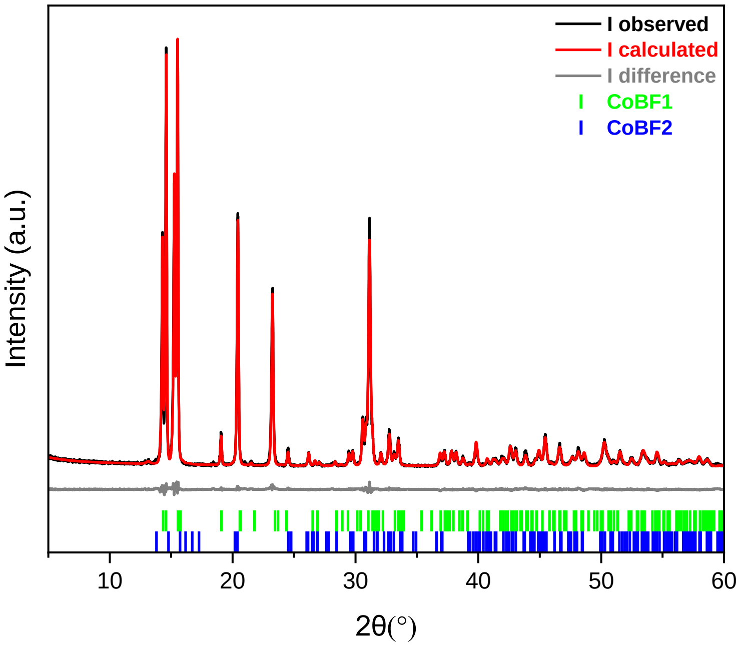

Figure S7. *Refinement results for CoBF method 3 sample. The experimental data is in black, and the calculated pattern is in red.* R_{wp}*=10.62%, GoF=1.4048. The vertical lines in different colours under the XRD pattern represent CoBF1 (in green), CoBF2 (in blue),*

Table S8. *Composition of the Sample CoBF method 3 obtained from the above refinement.*

Parameter	*Value*	*ESD*
CoBF1	*0.983*	*0.001*
CoBF2	*0.017*	*0.001*

2.7 CoBF 09/03

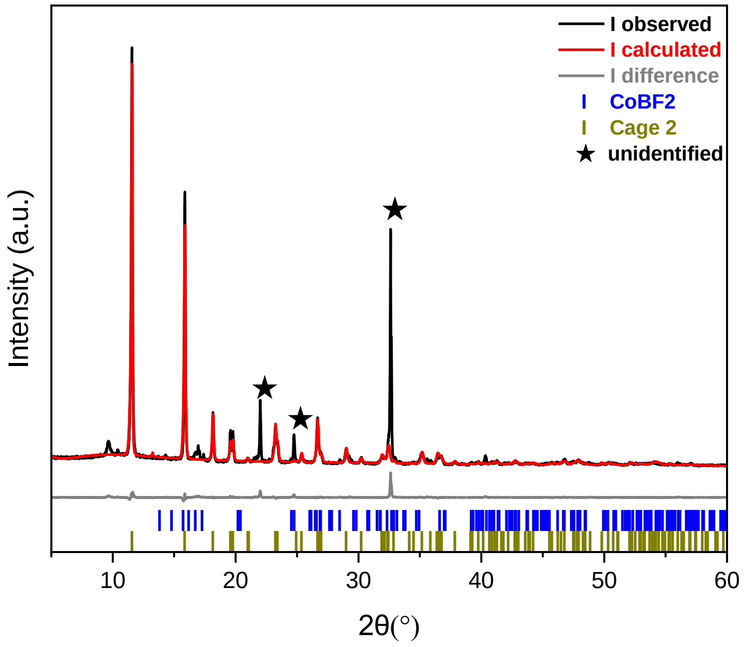

Figure S8. *Refinement result for CoBF 09/03 sample. The experimental data is in black, and the calculated pattern is in red. The peaks with★mark are from unidentified materials.* R_{wp}*=31.47%, GoF=4.7827. The vertical lines in different colours under the XRD pattern represent CoBF2 (in blue), Cage 2(in olive).*

Table S9. *Composition of the Sample CoBF 09/03 obtained from the above refinement. The peaks with★mark are calculated in the amorphous and unidentified part.*

Parameter	*Value*	*ESD*
CoBF2	*0.065*	*0.02*
Co Cage 2	*0.848*	*0.02*
Amorphous and unidentified (★)[a]	*0.087*	*0.005*

[a] *: The unidentified peaks (marked with ★) correspond to unknown phases for which crystallographic information could not be determined from our crystallisation experiments.*
Amorphous and unidentified %= total area of all Amorphous and unidentified peaks /total area of all crystalline and amorphous and unidentified peaks.

2.8 CoBF 09/06

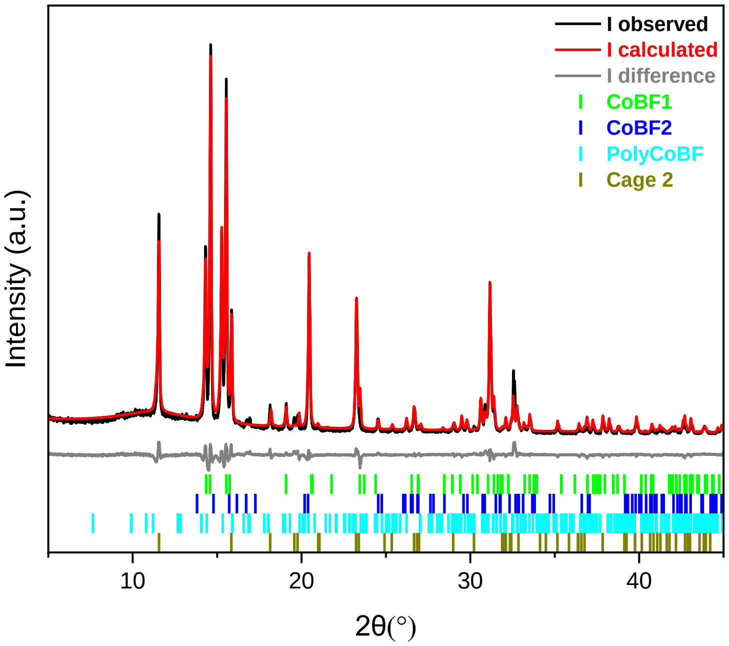

Figure S9. *Refinement result for CoBF 09/06 sample. The experimental data is in black, and the calculated pattern is in red. R_{wp}=16.05%, GoF=2.3194. The vertical lines in different colours under the XRD pattern represent CoBF1 (in green), CoBF2 (in blue), PolyCoBF (in cyan), Cage 2(in olive).*

Table S10. *Composition of the Sample CoBF 09/06 obtained from the above refinement.*

Parameter	*Value*	*ESD*
CoBF1	*0.686*	*0.007*
CoBF2	*0.154*	*0.006*
PolyCoBF	*0.012*	*0.003*
Co Cage 2	*0.148*	*0.004*

2.9 CoBF 10/01

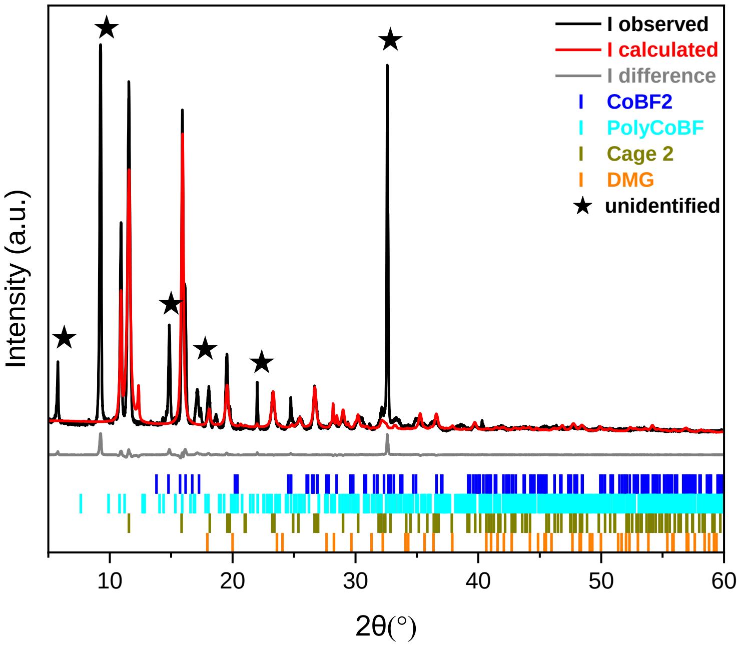

Figure S10. *Refinement result for CoBF 10/01 sample. The experimental data is in black, and the calculated pattern is in red. The peaks with★mark are from unidentified materials. R_{wp}=48.49%, GoF=8.7685. The vertical lines in different colours under the XRD pattern represent CoBF2 (in blue), PolyCoBF (in cyan), Cage 2(in olive), DMG (in orange).*
The refinement process did not reach a reasonable result due to the presence of unidentified phases. However, phase identification revealed that Co Cage2 is the predominant crystalline phase in the sample. The component composition was recalculated based on the quantities of amorphous and unidentified phases, as presented in Table S11.

Table S11.*Composition of the Sample CoBF 10/01 obtained from the above refinement. The peaks with★mark are calculated in the amorphous and unidentified part.*

Parameter	*Value*	*ESD*
CoBF2	*0.0551*	*0.0100*
PolyCoBF	*0.0206*	*0.0070*
Co Cage 2	*0.608*	*0.020*
DMG	*0.1134*	*0.009*
Amorphous and unidentified(★)[a]	*0.2029*	*/*

[a] *: The unidentified peaks (marked with ★) correspond to unknown phases for which crystallographic information could not be determined from our crystallisation experiments.*
Amorphous and unidentified %= total area of all Amorphous and unidentified peaks /total area of all crystalline and amorphous and unidentified peaks.

2.10 BW CoBF 1

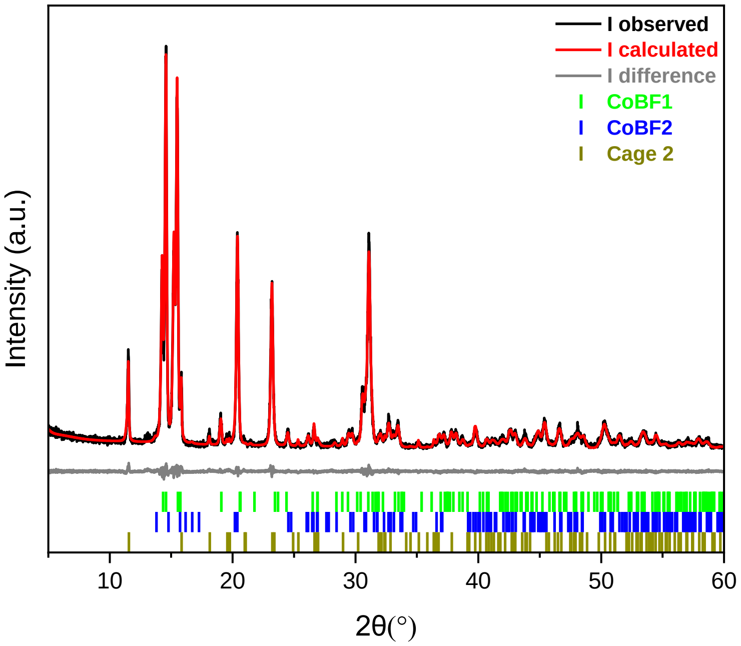

Figure S11. *Refinement result for BW CoBF 1 sample. The experimental data is in black, and the calculated pattern is in red.* R_{wp}*=14.53%, GoF=1.2169. The vertical lines in different colours under the XRD pattern represent CoBF1 (in green), CoBF2 (in blue), Cage 2(in olive).*

Table S12. *Composition of the Sample BW CoBF 1 obtained from the above refinement.*

Parameter	*Value*	*ESD*
CoBF1	*0.919*	*0.003*
CoBF2	*0.021*	*0.003*
Co Cage 2	*0.06*	*0.003*

2.11 BW CoBF 2

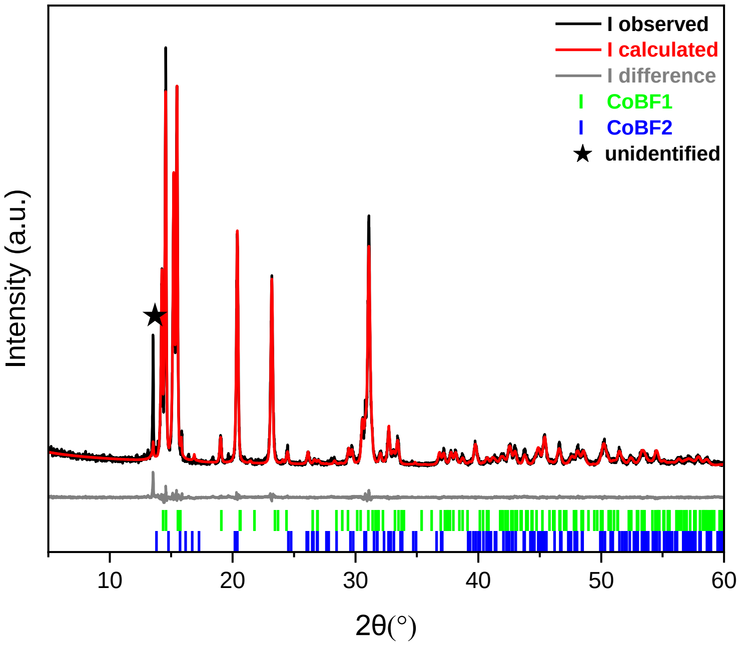

Figure S12. *Refinement result for BW CoBF 2 sample. The experimental data is in black, and the calculated pattern is in red. The peaks with★mark are from unidentified materials.* R_{wp}*=17.45%, GoF=1.4627. The vertical lines in different colours under the XRD pattern represent CoBF1 (in green), CoBF2 (in blue).*

Table S13. *Composition of the Sample BW CoBF 2 obtained from the above refinement. The peaks with★mark are calculated in the amorphous and unidentified part.*

Parameter	*Value*	*ESD*
CoBF1	*0.884*	*0.004*
CoBF2	*0.088*	*0.004*
Amorphous and unidentified (★)[a]	*0.028*	*/*

[a] *: The unidentified peaks (marked with ★) correspond to unknown phases for which crystallographic information could not be determined from our crystallisation experiments.*
Amorphous and unidentified %= total area of all Amorphous and unidentified peaks /total area of all crystalline and amorphous and unidentified peaks.

2.12 Strem CoBF

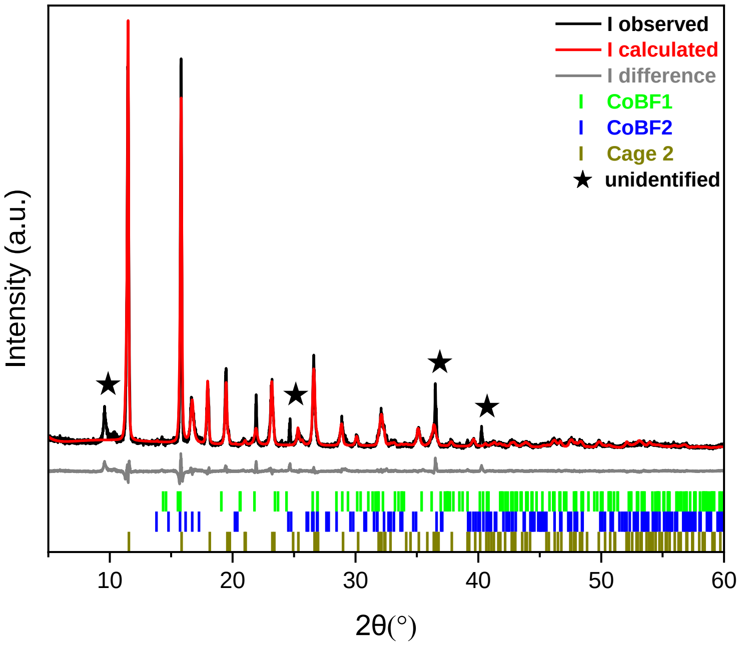

Figure S13. *Refinement result for Strem CoBF sample. The experimental data is in black, and the calculated pattern is in red. The peaks with★mark are from unidentified materials.* R_{wp}*=22.86%, GoF=1.8846. The vertical lines in different colours under the XRD pattern represent CoBF1 (in green), CoBF2 (in blue), Cage 2(in olive). The component composition was recalculated according to the amount of the amorphous and unidentified shown in Table S14.*

Table S14. *Composition of the Sample Strem CoBF obtained from the above refinement. The peaks with★mark are calculated in the amorphous and unidentified part.*

Parameter	*Value*	*ESD*
CoBF1	*0.133*	*0.006*
CoBF2	*0.0029*	*0.001*
Co Cage 2	*0.836*	*0.019*
Amorphous and unidentified (★)[a]	*0.028*	*/*

[a] *: The unidentified peaks (marked with ★) correspond to unknown phases for which crystallographic information could not be determined from our crystallisation experiments.*

Amorphous and unidentified %= total area of all Amorphous and unidentified peaks /total area of all crystalline and amorphous and unidentified peaks.

3 Bulk polymerization of MMA with different CoBF samples

3.1 CoBF 1998

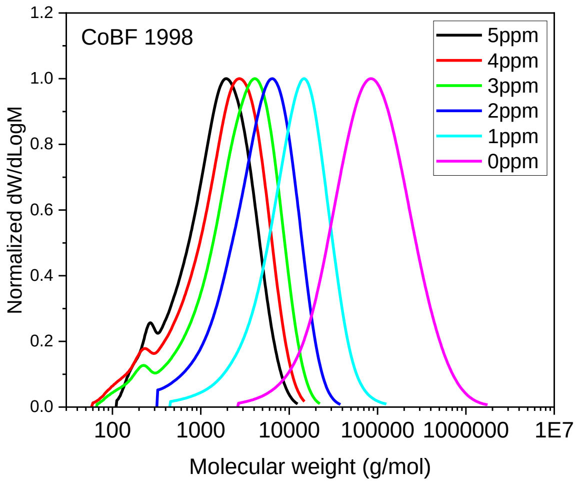

Figure S14. *GPC traces of PMMA made with CoBF 1998 polymerized in the absence of solvent at 70°C with 0.25 wt% AIBN for 10 minutes.*

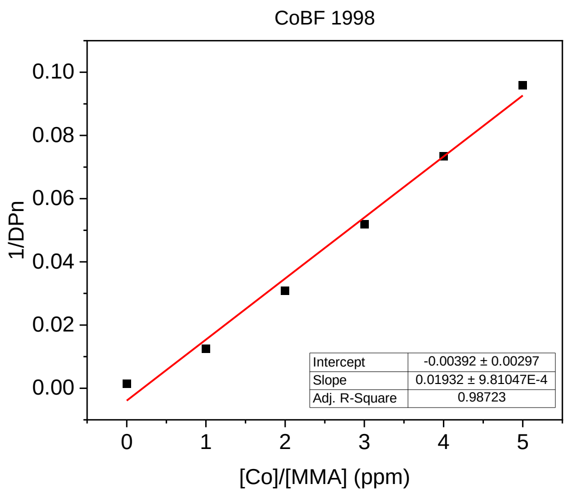

Figure S15. *Mayo plot for polymerization of MMA using CoBF 1998.*

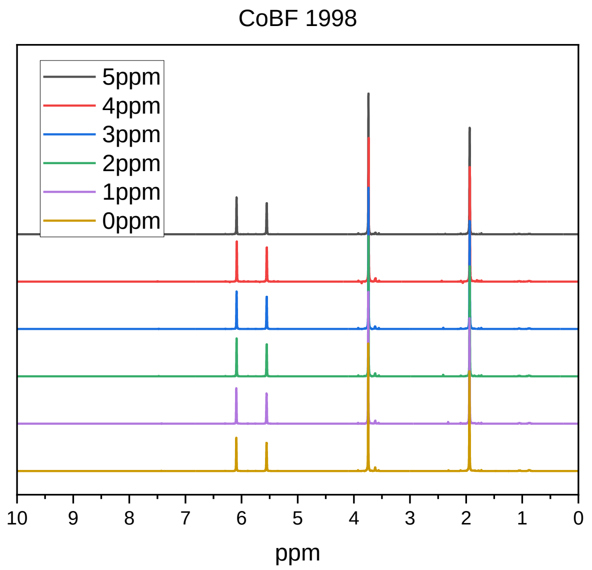

Figure S16. *^{1}H NMR (400 MHz) of PMMA made with CoBF 1998 in $CDCl_3$.*

Table S15. *Effect of changing the [CoBF] ppm on the molecular weight, dispersity and monomer conversion of the resulting PMMA with CoBF 1998.*

ppm	M_n (g/mol)	M_w (g/mol)	1/DPn	Đ	Conversion%
0	*50800*	*130000*	*0.00153528*	*2.57*	*4.6*
1	*7380*	*16000*	*0.012545705*	*2.16*	*4.2*
2	*3110*	*6490*	*0.030858684*	*2.09*	*4.0*
3	*1290*	*3860*	*0.051929979*	*3.00*	*3.6*
4	*953*	*2730*	*0.07342941*	*2.86*	*2.9*
5	*941*	*2090*	*0.095947293*	*2.22*	*2.8*

3.2 CoBF 1999

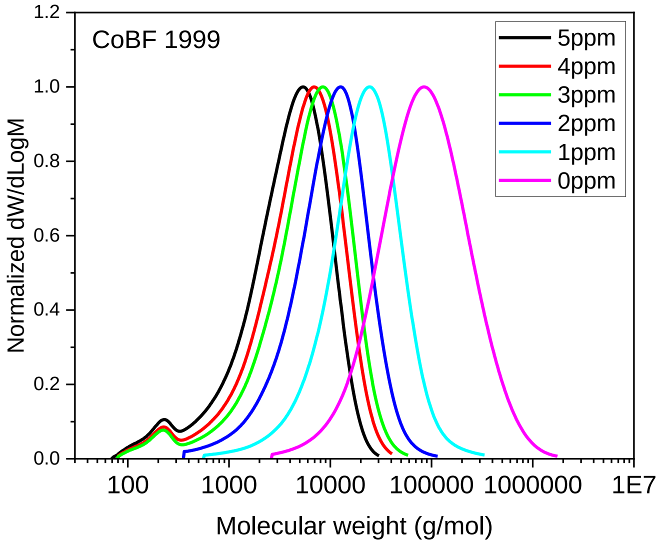

Figure S17. *GPC traces of PMMA made with CoBF 1999 polymerized in the absence of solvent at 70°C with 0.25 wt% AIBN for 10 minutes.*

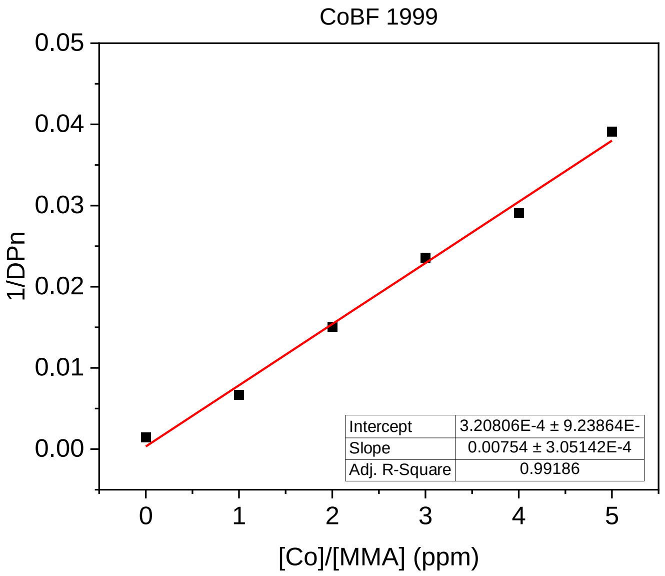

Figure S18. *Mayo plot for polymerization of MMA using CoBF 1999.*

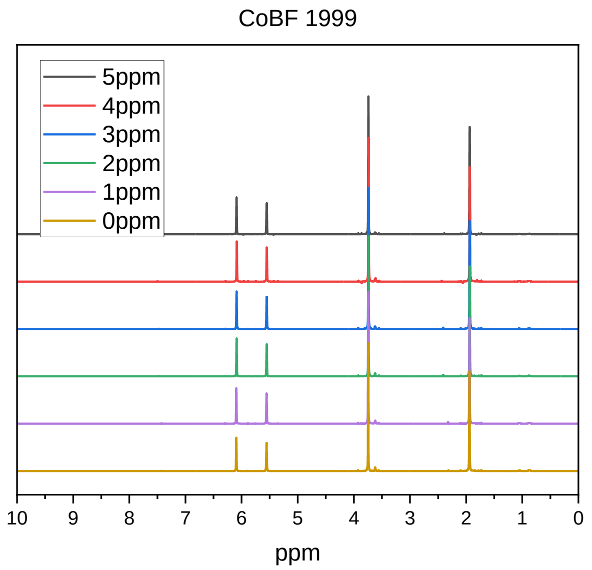

Figure S19. *^{1}H NMR (400 MHz) of PMMA made with CoBF 1999 in $CDCl_3$.*

Table S16. *Effect of changing the [CoBF] ppm on the molecular weight, dispersity and monomer conversion of the resulting PMMA with CoBF 1999.*

ppm	M_n (g/mol)	M_w (g/mol)	1/DPn	Đ	Conversion%
0	*50800*	*130000*	*0.00153528*	*2.569*	*4.6*
1	*13200*	*30000*	*0.006680523*	*2.277*	*4.2*
2	*5950*	*13300*	*0.015060319*	*2.233*	*4.2*
3	*2430*	*8490*	*0.02358563*	*3.488*	*3.8*
4	*2094*	*6894*	*0.029045837*	*3.292*	*3.0*
5	*1641*	*5120*	*0.039109766*	*3.12*	*2.6*

3.3 CoBF Ark

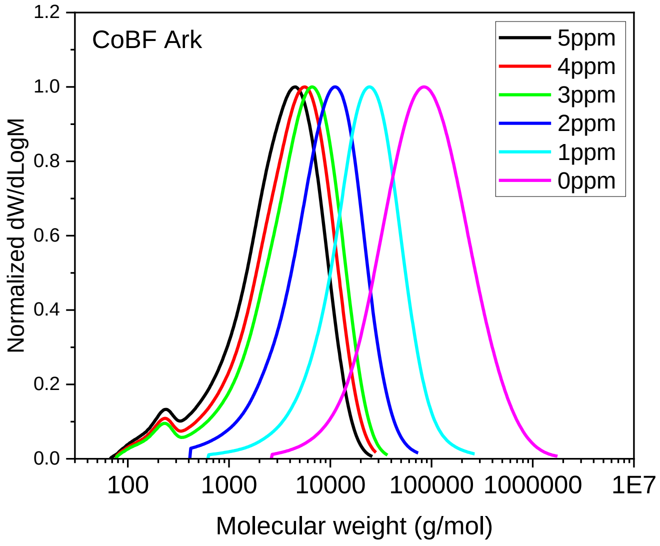

Figure S20. *GPC traces of PMMA made with CoBF Ark polymerized in the absence of solvent at 70°C with 0.25 wt% AIBN for 10 minutes.*

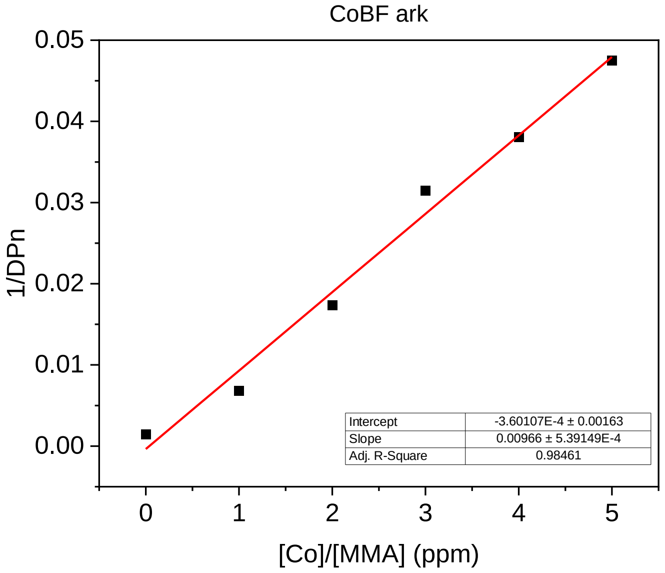

Figure S21. *Mayo plot for polymerization of MMA using CoBF Ark.*

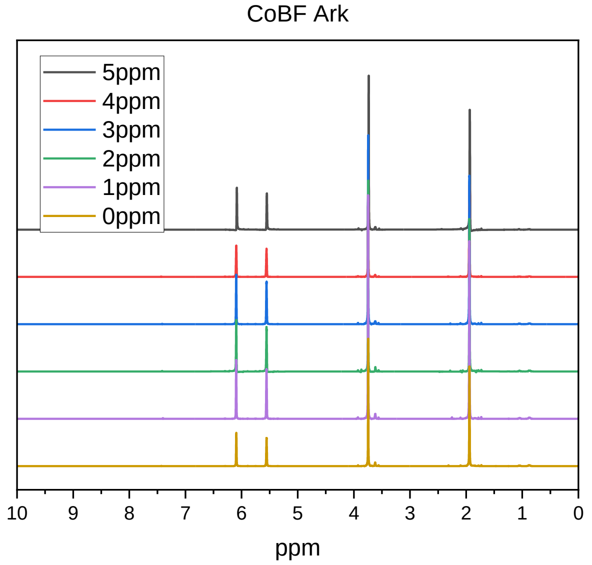

Figure S22. *^{1}H NMR (400 MHz) traces of PMMA made with CoBF Ark in $CDCl_3$.*

Table S17. *Effect of changing the [CoBF] ppm on the molecular weight, dispersity and monomer conversion of the resulting PMMA with CoBF Ark.*

ppm	M_n (g/mol)	M_w (g/mol)	1/DPn	Đ	Conversion%
0	*50800*	*130000*	*0.00153528*	*2.57*	*4.6*
1	*13200*	*29300*	*0.006834198*	*2.22*	*4.0*
2	*5340*	*11500*	*0.017342976*	*2.16*	*3.0*
3	*1930*	*6370*	*0.031454917*	*3.30*	*3.3*
4	*1660*	*5260*	*0.038047121*	*3.16*	*3.1*
5	*1380*	*4210*	*0.047529551*	*3.06*	*3.2*

3.4 CoBF method 1

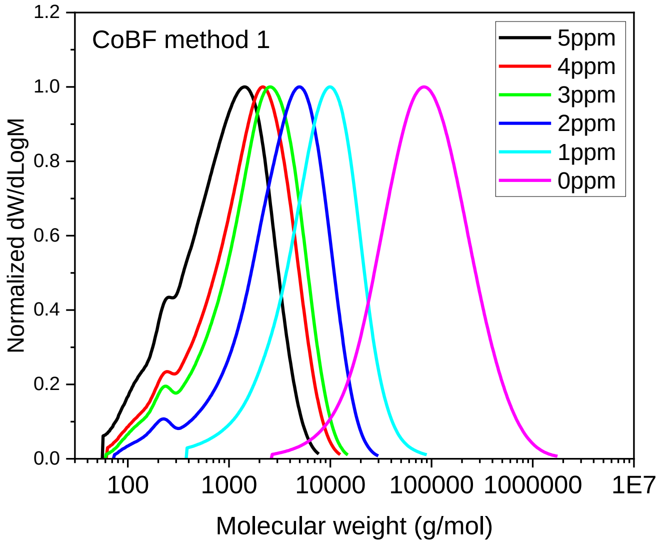

Figure S23. *GPC traces of PMMA made with CoBF method 1 polymerized in the absence of solvent at 70°C with 0.25 wt% AIBN for 10 minutes.*

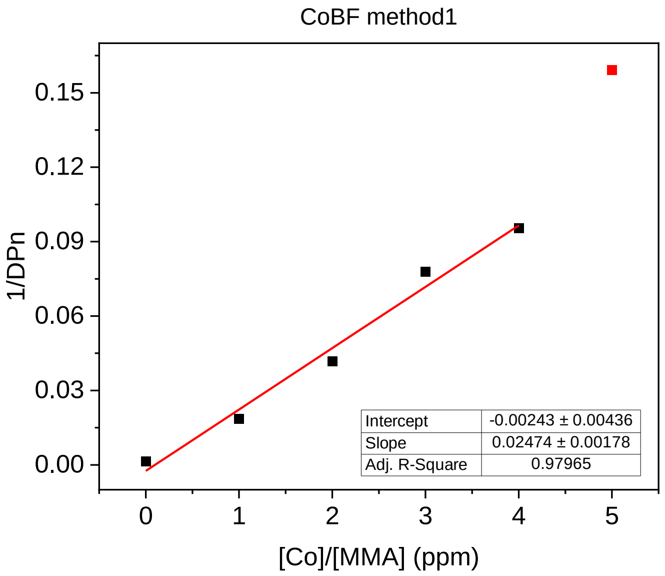

Figure S24. *Mayo plot for polymerization of MMA using CoBF method 1.*

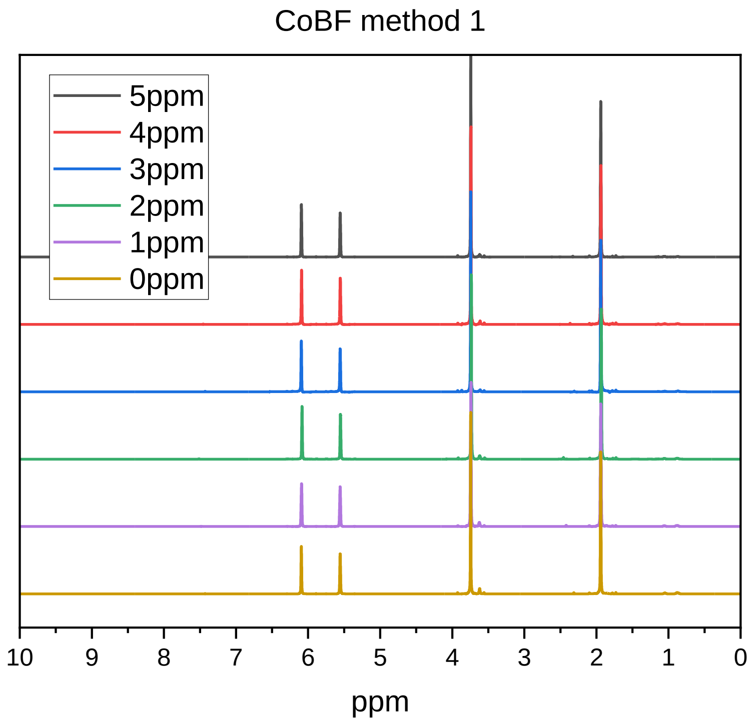

Figure S25. *^{1}H NMR (400 MHz) of PMMA made with CoBF method 1 in $CDCl_3$.*

Table S18. *Effect of changing the [CoBF] ppm on the molecular weight, dispersity and monomer conversion of the resulting PMMA with CoBF method 1.*

ppm	M_n (g/mol)	M_w (g/mol)	1/DPn	Đ	Conversion%
0	50800	130000	0.00153528	2.57	4.6
1	4900	10800	0.018508365	2.22	4.4
2	1540	4780	0.041865356	3.10	3.9
3	915	2570	0.07788487	2.81	3.0
4	763	2100	0.095489747	2.75	3.2
5	496	1260	0.159174881	2.54	3.3

3.5 CoBF method 2

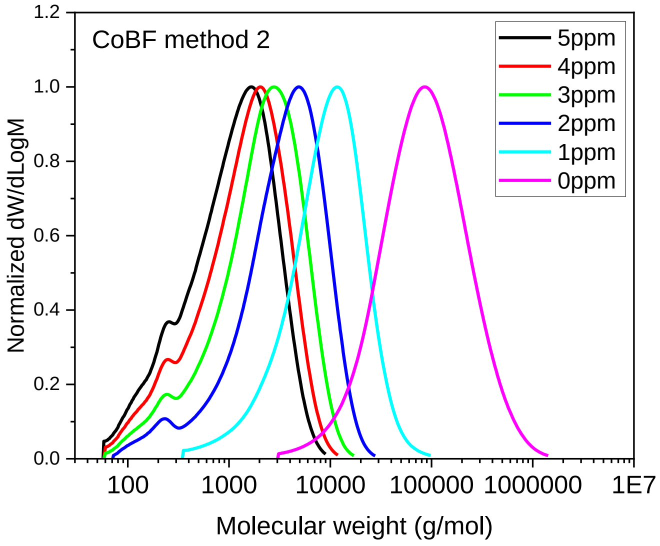

Figure S26. *GPC traces of PMMA made with CoBF method 2 polymerized in the absence of solvent at 70°C with 0.25 wt% AIBN for 10 minutes.*

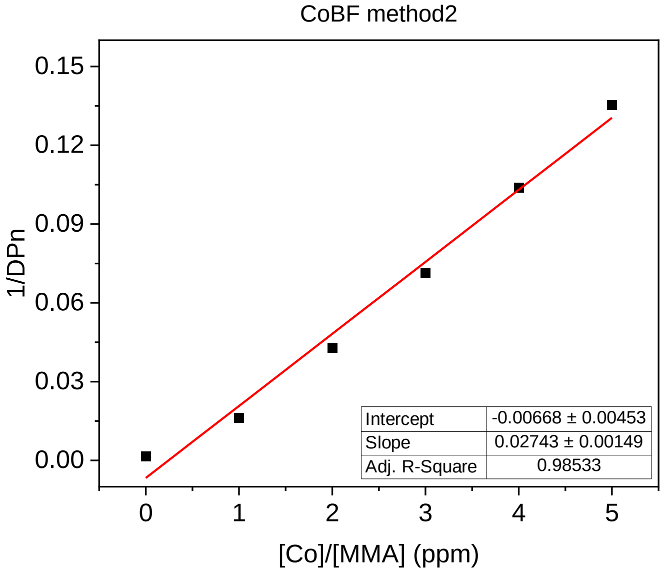

Figure S27. *Mayo plot for polymerization of MMA using CoBF method 2.*

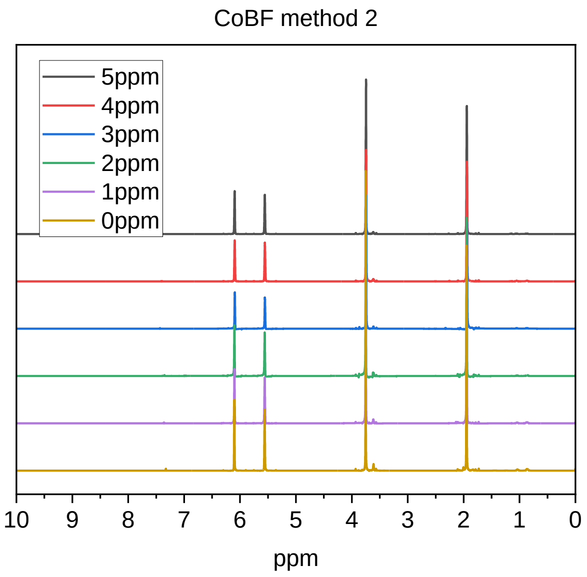

Figure S28. *^{1}H NMR (400 MHz) of PMMA made with CoBF method 2 in $CDCl_3$.*

Table S19. *Effect of changing the [CoBF] ppm on the molecular weight, dispersity and monomer conversion of the resulting PMMA with CoBF method 2.*

**ppm**	**M_n (g/mol)**	**M_w (g/mol)**	**1/DPn**	**Đ**	**Conversion%**
0	*50800*	*130000*	*0.00153528*	*2.57*	*4.6*
1	*5490*	*12300*	*0.016299715*	*2.24*	*3.3*
2	*1520*	*4670*	*0.042915131*	*3.07*	*3.4*
3	*976*	*2800*	*0.071438459*	*2.87*	*3.1*
4	*704*	*1930*	*0.103859959*	*2.74*	*3.1*
5	*567*	*1480*	*0.135298649*	*2.61*	*3.0*

3.6 CoBF method 3

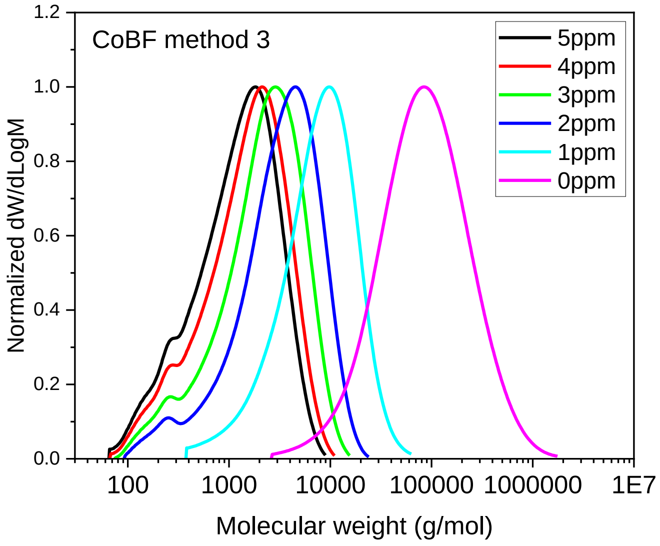

Figure S29. *GPC traces of PMMA made with CoBF method 3 polymerized in the absence of solvent at 70°C with 0.25 wt% AIBN for 10 minutes.*

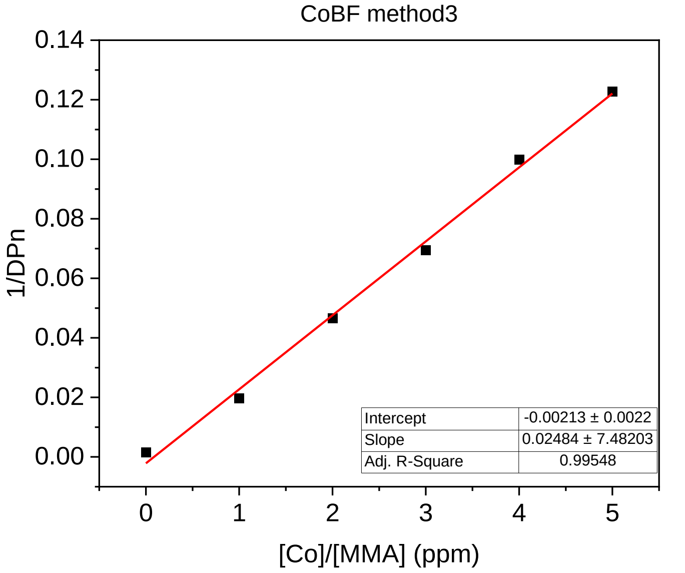

Figure S30. *Mayo plot for polymerization of MMA using CoBF method 3.*

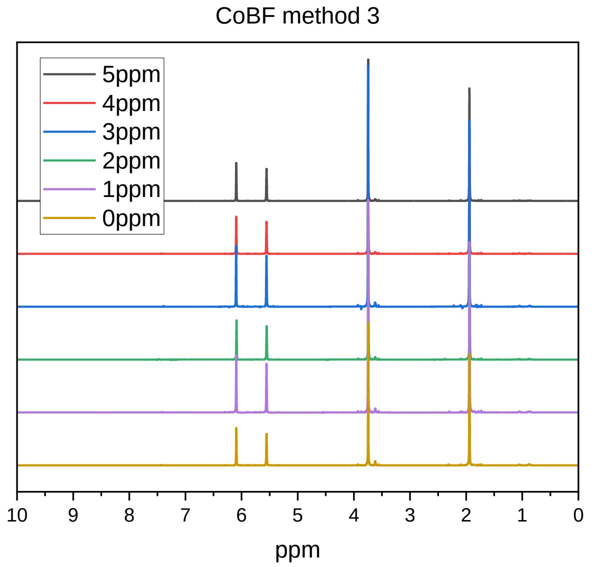

Figure S31. *^{1}H NMR (400 MHz) of PMMA made with CoBF method 3 in $CDCl_3$.*

Table S20. *Effect of changing the [CoBF] ppm on the molecular weight, dispersity and monomer conversion of the resulting PMMA with CoBF method 3.*

ppm	M_n (g/mol)	M_w (g/mol)	1/DPn	Đ	Conversion%
0	*48800*	*135000*	*0.001482253*	*2.77*	*5.0*
1	*4800*	*10100*	*0.019741891*	*2.11*	*3.7*
2	*1580*	*4300*	*0.046578739*	*2.72*	*3.9*
3	*1108*	*2880*	*0.069432039*	*2.60*	*4.0*
4	*804*	*2000*	*0.099921158*	*2.49*	*3.5*
5	*676*	*1630*	*0.122697304*	*2.41*	*3.6*

3.7 CoBF 09/03

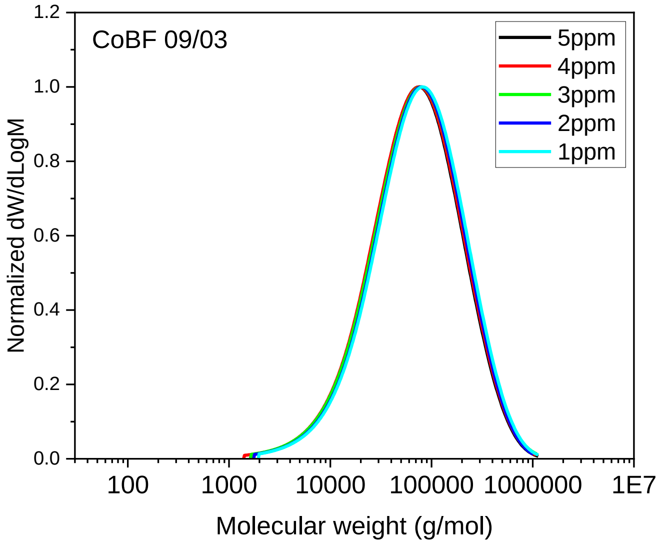

Figure S32. *GPC traces of PMMA made with CoBF 09/03 polymerized in the absence of solvent at 70°C with 0.25 wt% AIBN for 10 minutes.*

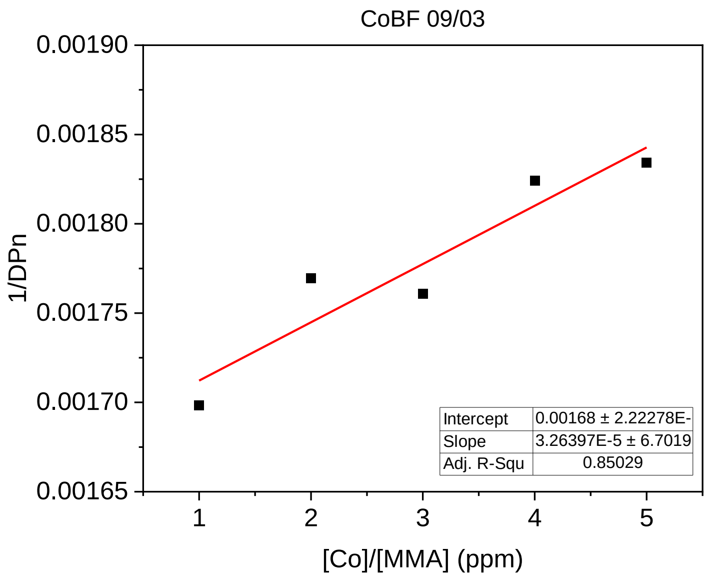

Figure S33. *Mayo plot for polymerization of MMA using CoBF 09/03.*

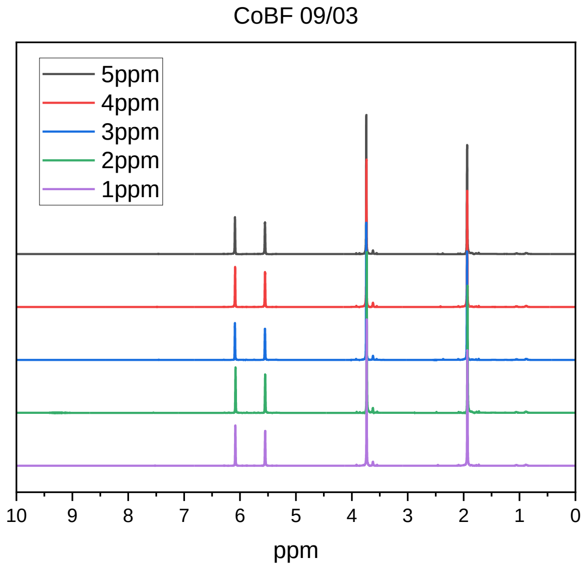

Figure S34. *^{1}H NMR (400 MHz) of PMMA made with CoBF 09/03 in $CDCl_3$.*

Table S21. *Effect of changing the [CoBF] ppm on the molecular weight, dispersity and monomer conversion of the resulting PMMA with CoBF 09/03.*

ppm	M_n (g/mol)	M_w (g/mol)	1/DPn	Đ	Conversion%
1	*40600*	*118000*	*0.001699*	*2.90*	*4.8*
2	*39200*	*113000*	*0.00177*	*2.88*	*4.5*
3	*37800*	*114000*	*0.001761*	*3.01*	*5.0*
4	*36700*	*110000*	*0.001824*	*2.99*	*4.7*
5	*37700*	*109000*	*0.001834*	*2.89*	*4.7*

3.8 CoBF 09/06

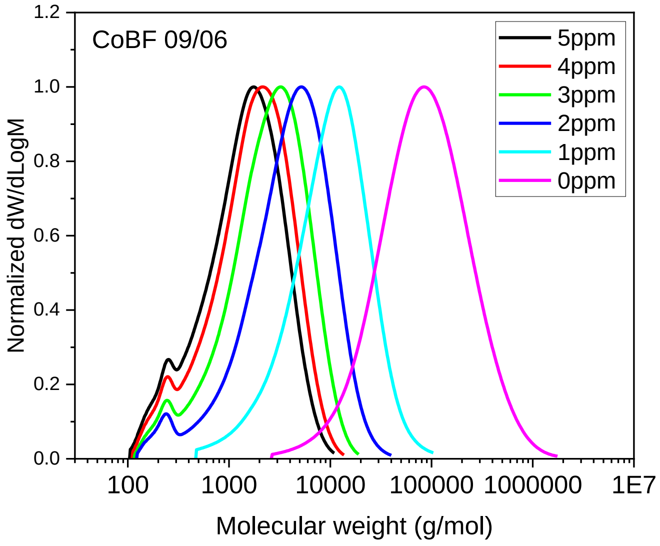

Figure S35. *GPC traces of PMMA made with CoBF 09/06 polymerized in the absence of solvent at 70°C with 0.25 wt% AIBN for 10 minutes.*

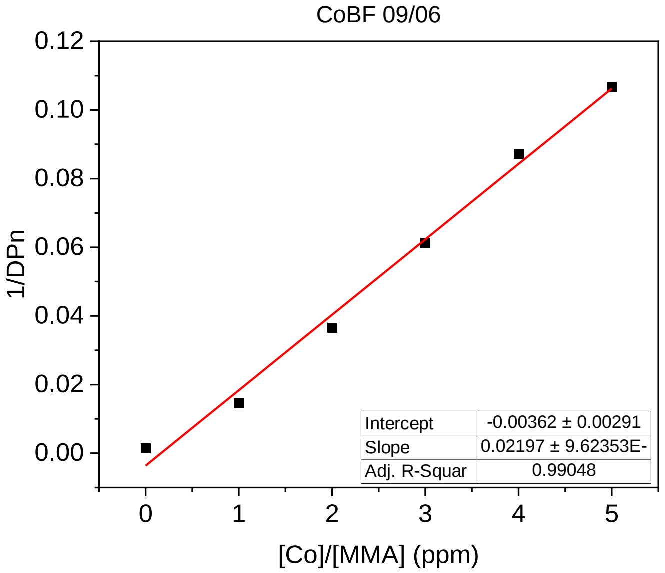

Figure S36. *Mayo plot for polymerization of MMA using CoBF 09/06.*

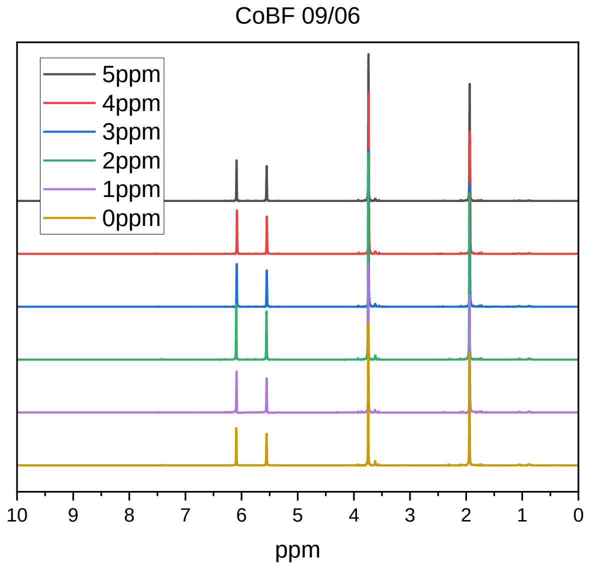

Figure S37. *^{1}H NMR (400 MHz) of PMMA made with CoBF 09/06 in $CDCl_3$.*

Table S22. *Effect of changing the [CoBF] ppm on the molecular weight, dispersity and monomer conversion of the resulting PMMA with CoBF 09/06.*

ppm	M_n (g/mol)	M_w (g/mol)	1/DPn	Đ	Conversion%
0	*50800*	*130000*	*0.00153528*	*2.57*	*4.6*
1	*6171*	*13700*	*0.014607674*	*2.22*	*3.1*
2	*1932*	*5490*	*0.036500547*	*2.84*	*3.6*
3	*1343*	*3270*	*0.061292317*	*2.43*	*4.1*
4	*1006*	*2290*	*0.087251416*	*2.28*	*3.6*
5	*850*	*1880*	*0.106738806*	*2.21*	*3.3*

3.9 CoBF 10/01

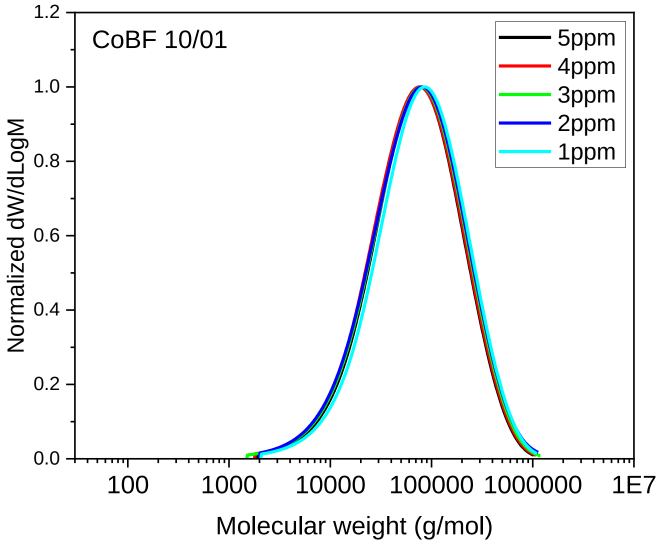

Figure S38. *GPC traces of PMMA made with CoBF 10/01 polymerized in the absence of solvent at 70°C with 0.25 wt% AIBN for 10 minutes.*

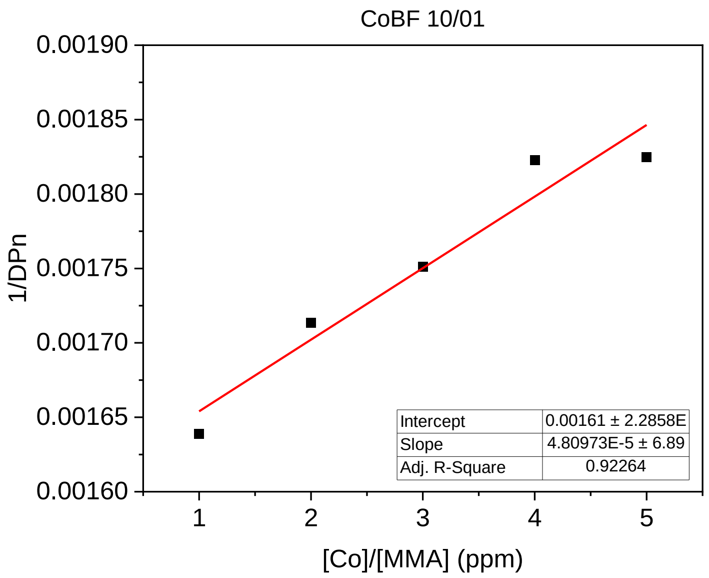

Figure S39. *Mayo plot for polymerization of MMA using CoBF 10/01.*

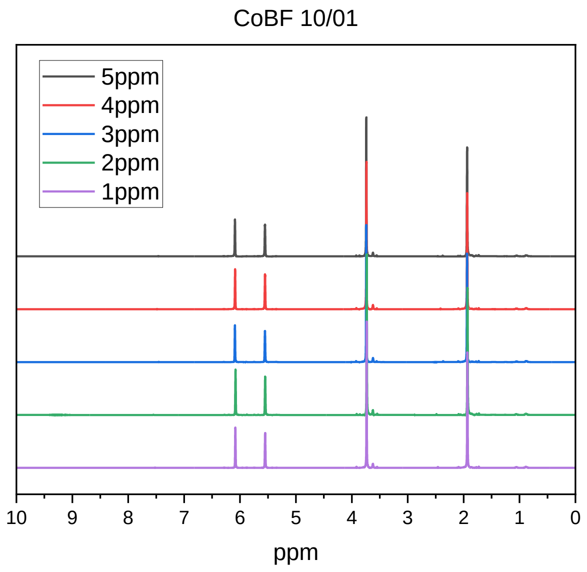

Figure S40. *^{1}H NMR (400 MHz) of PMMA made with CoBF 10/01 in $CDCl_3$.*

Table S23. *Effect of changing the [CoBF] ppm on the molecular weight, dispersity and monomer conversion of the resulting PMMA with CoBF 10/01.*

ppm	Mn (g/mol)	Mw (g/mol)	1/DPn	Đ	Conversion%
1	*42900*	*122000*	*0.001638856*	*2.84*	*4.3*
2	*38500*	*117000*	*0.001713506*	*3.03*	*4.5*
3	*37700*	*114000*	*0.00175127*	*3.03*	*4.9*
4	*37400*	*110000*	*0.001822702*	*2.94*	*4.9*
5	*38800*	*110000*	*0.001824745*	*2.83*	*4.6*

3.10 BW CoBF 1

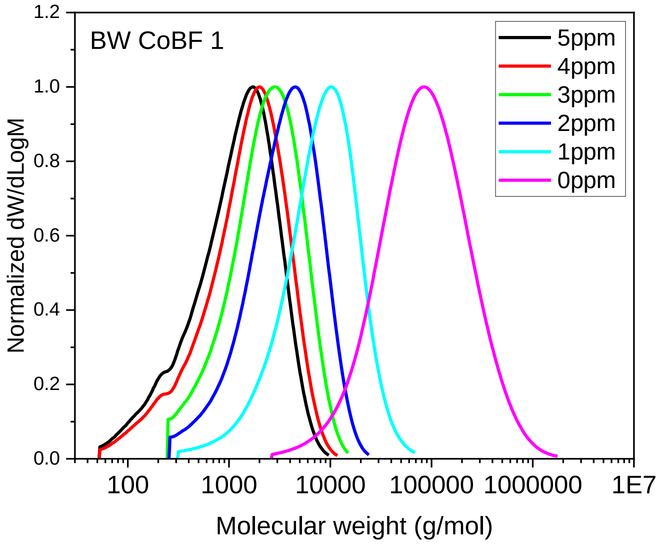

Figure S41. *GPC traces of PMMA made with BW CoBF 1 polymerized in the absence of solvent at 70°C with 0.25 wt% AIBN for 10 minutes.*

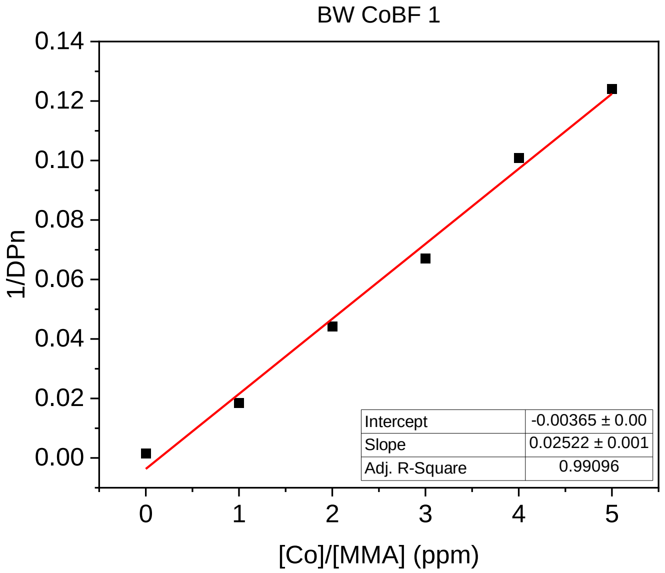

Figure S42. *Mayo plot for polymerization of MMA using BW CoBF 1.*

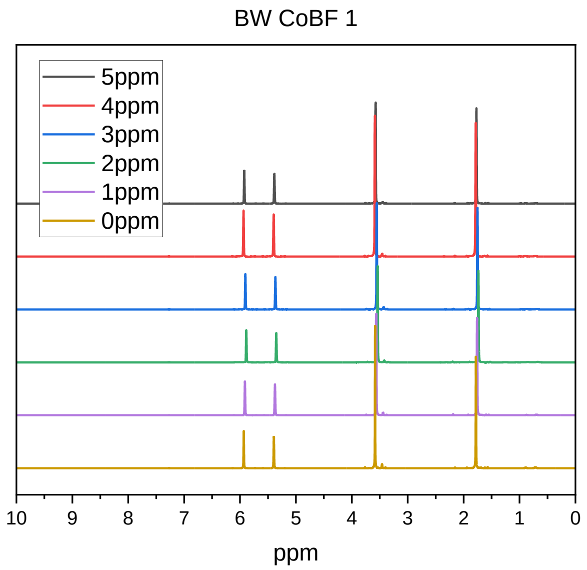

Figure S43. *^{1}H NMR (400 MHz) of PMMA made with BW CoBF 1 in $CDCl_3$.*

Table S24. *Effect of changing the [CoBF] ppm on the molecular weight, dispersity and monomer conversion of the resulting PMMA with BW CoBF 1.*

ppm	M_n (g/mol)	M_w (g/mol)	1/DPn	Đ	Conversion%
0	*50800*	*130000*	*0.00153528*	*2.57*	*4.6*
1	*5020*	*10800*	*0.018585669*	*2.14*	*3.4*
2	*2270*	*4530*	*0.044213292*	*1.99*	*3.0*
3	*1570*	*2980*	*0.067150235*	*1.90*	*3.6*
4	*760*	*1990*	*0.100826788*	*2.61*	*3.1*
5	*640*	*1610*	*0.124065675*	*2.54*	*2.8*

3.11 BW CoBF 2

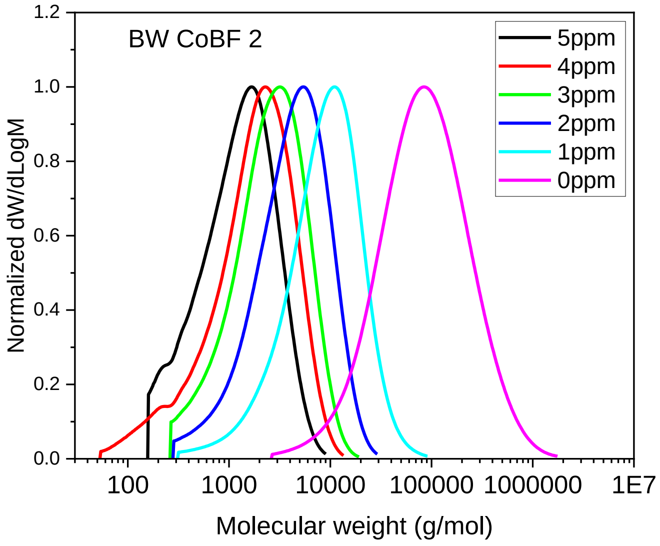

Figure S44. *GPC traces of PMMA made with BW CoBF 2 polymerized in the absence of solvent at 70°C with 0.25 wt% AIBN for 10 minutes.*

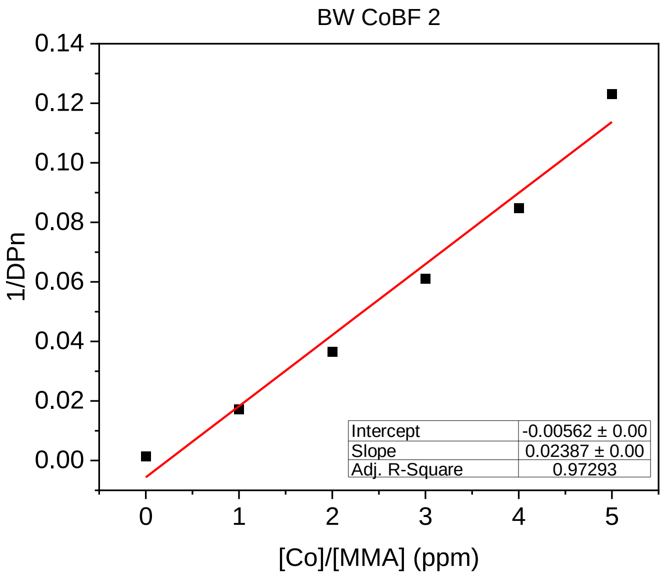

Figure S45. *Mayo plot for polymerization of MMA using BW CoBF 2.*

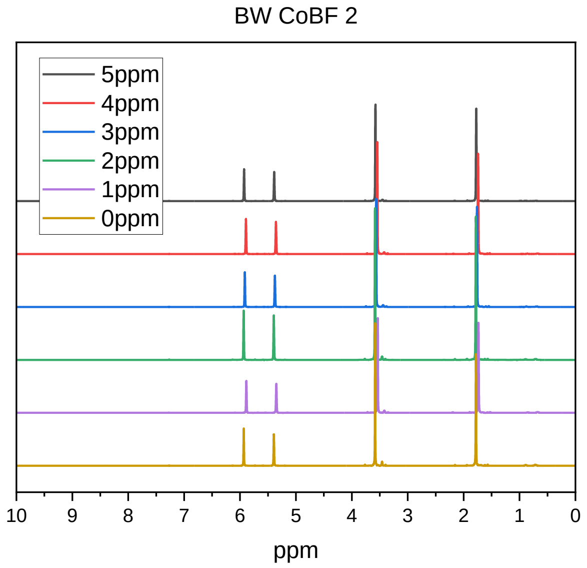

Figure S46. *^{1}H NMR (400 MHz) of PMMA made with BW CoBF 2 in $CDCl_3$.*

Table S25. *Effect of changing the [CoBF] ppm on the molecular weight, dispersity and monomer conversion of the resulting PMMA with BW CoBF 2.*

ppm	M_n (g/mol)	M_w (g/mol)	1/DPn	Đ	Conversion%
0	*50800*	*130500*	*0.00153528*	*2.57*	*4.6*
1	*5350*	*11600*	*0.017260753*	*2.17*	*3.6*
2	*2710*	*5480*	*0.036567202*	*2.02*	*3.5*
3	*1710*	*3280*	*0.061105279*	*1.91*	*3.2*
4	*880*	*2360*	*0.084776461*	*2.67*	*2.9*
5	*830*	*1630*	*0.123150062*	*1.97*	*2.7*

3.12 Strem CoBF

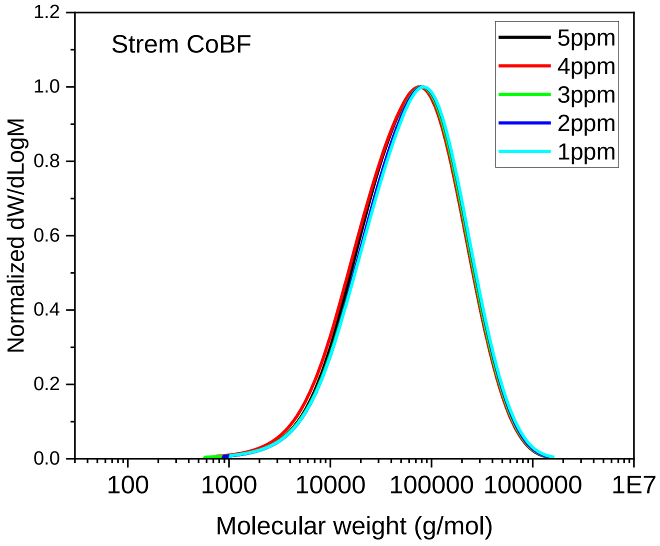

Figure S47. *GPC traces of PMMA made with Strem CoBF polymerized in the absence of solvent at 70°C with 0.25 wt% AIBN for 10 minutes.*

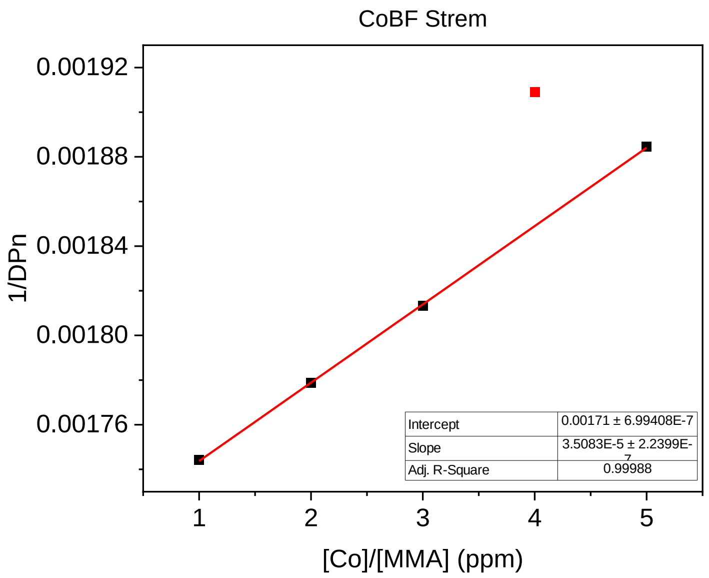

Figure S48. *Mayo plot for polymerization of MMA using Strem CoBF.*

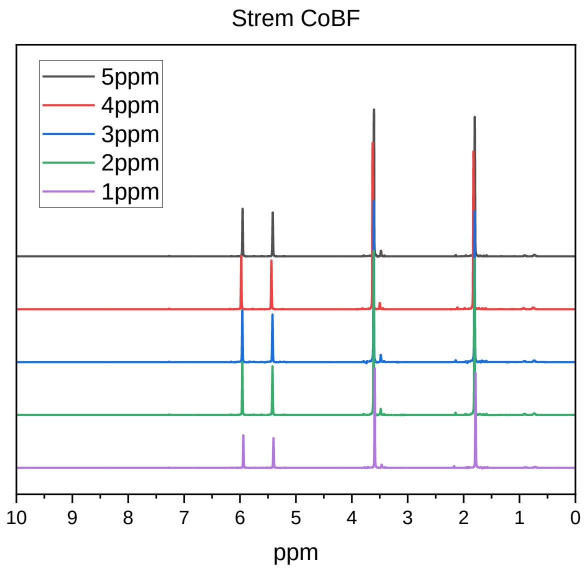

Figure S49. *^{1}H NMR (400 MHz) of PMMA made with Strem CoBF in $CDCl_3$.*

Table S26. *Effect of changing the [CoBF] ppm on the molecular weight, dispersity and monomer conversion of the resulting PMMA with Strem CoBF.*

ppm	M_n (g/mol)	M_w (g/mol)	1/DPn	Đ	Conversion%
1	*30700*	*115000*	*0.001744*	*3.74*	*4.6*
2	*30300*	*113000*	*0.001779*	*3.72*	*4.7*
3	*28800*	*110000*	*0.001813*	*3.84*	*4.7*
4	*26800*	*105000*	*0.001909*	*3.91*	*4.8*
5	*29200*	*106000*	*0.001884*	*3.64*	*4.8*

4. Infrared spectrum

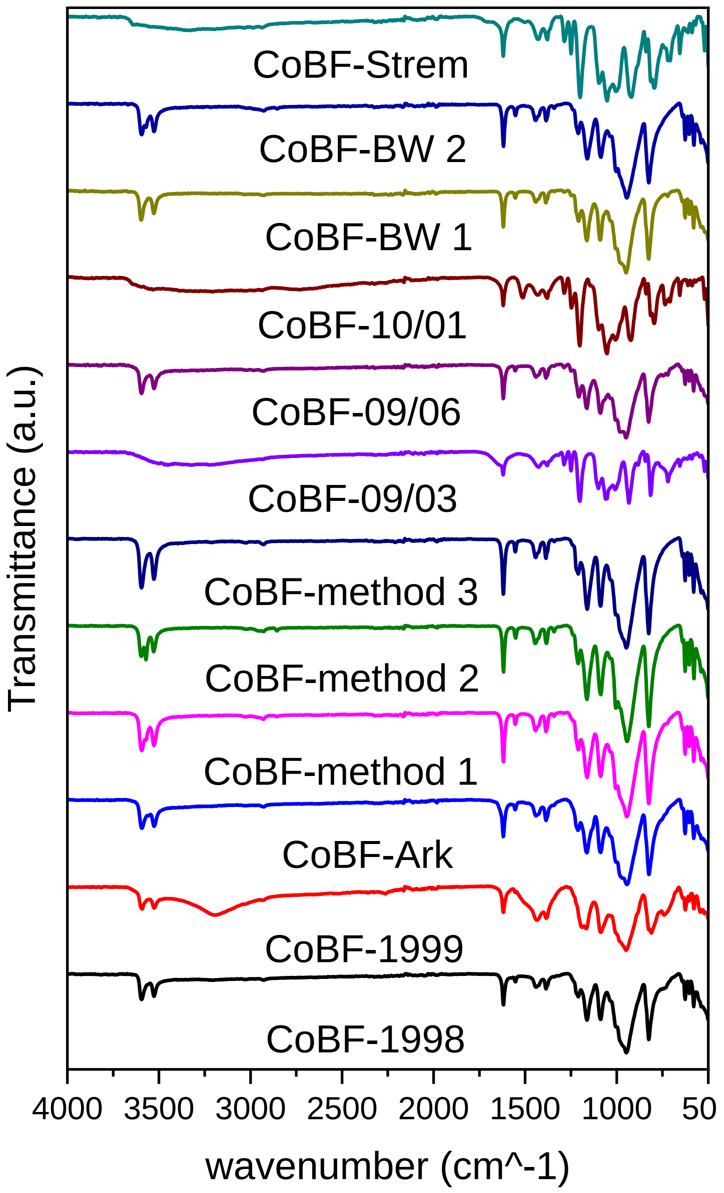

Figure S50. *Normalized Infrared spectrum of CoBF powder samples from 400 cm^{-1} to 4000 cm^{-1}.*

5. X-ray photoelectron spectroscopy

5.1 Cobalt(II) chloride hexahydrate

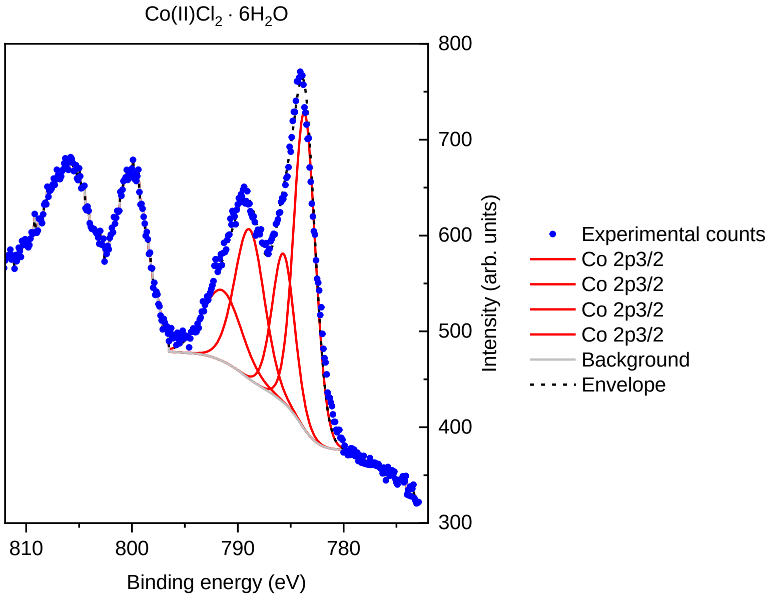

Figure S51. *Co 2p spectra of Cobalt(II) chloride hexahydrate.*

5.2 Cobalt(III) acetylacetonate

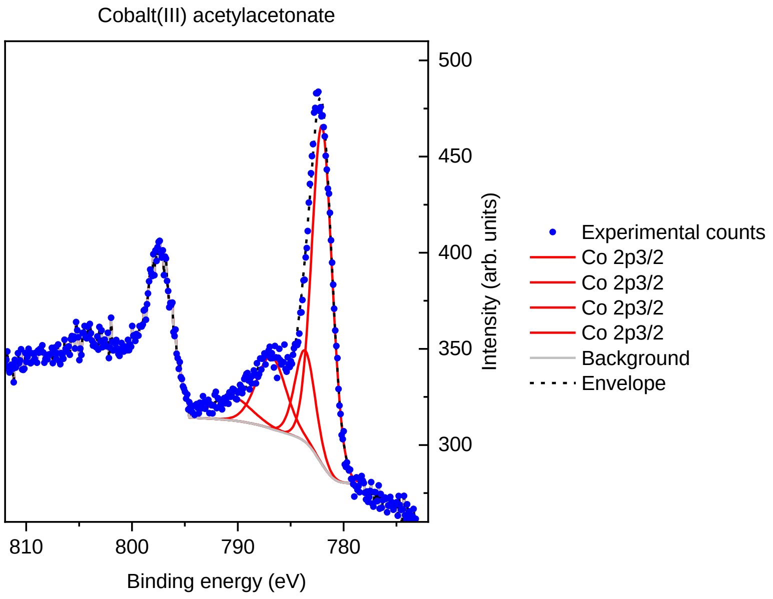

Figure S52. *Co 2p spectra of Cobalt(III) acetylacetonate.*

5.3 Chloro(pyridine)bis(dimethylglyoximato)cobalt(III)

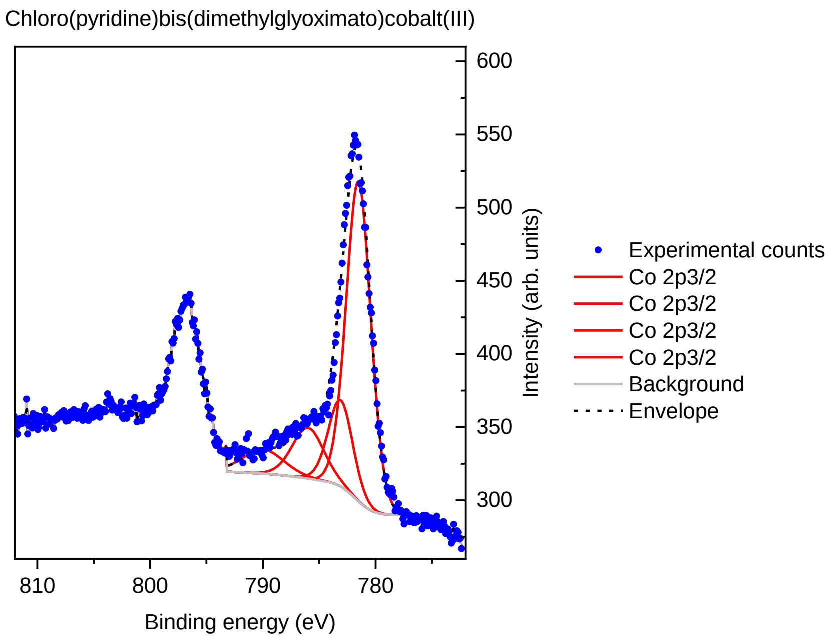

Figure S53. *Co 2p spectra of Chloro(pyridine)bis(dimethylglyoximato)cobalt(III).*

5.4 PolyCoBF

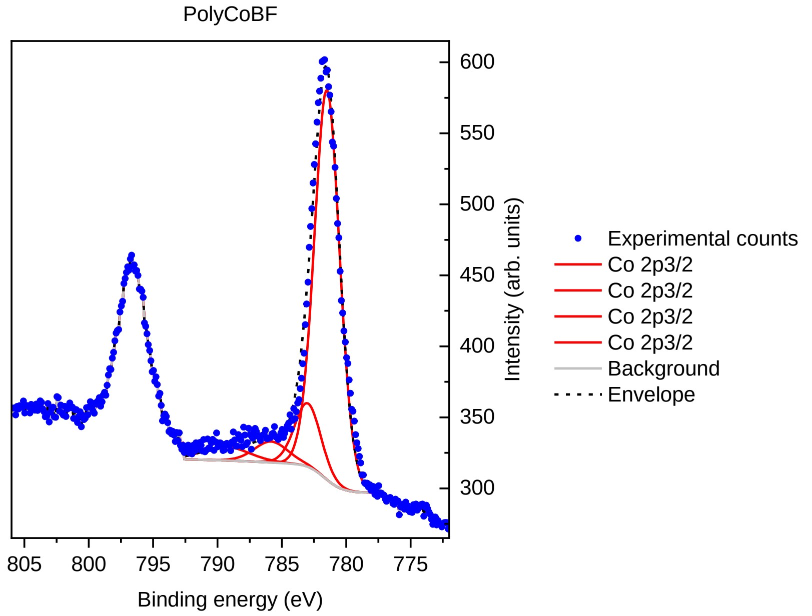

Figure S54. *Co 2p spectra of PolyCoBF.*

References

[1] E. C. Lingafelter, G. A. Zakrzewski, and C. A. Ghilardi. Journal of the American Chemical Society. 1971, 93 (18), 4411-4415.

[2] M. Sakata and M. J. Cooper J. Appl. Cryst. (1979). 12, 554-563

[3] N. Döbelin, R. Kleeberg, Journal of Applied Crystallography **2015***, 48, 1573-1580*
